# Clinical genomic profiling in the management of patients with soft tissue and bone sarcoma

**DOI:** 10.1038/s41467-022-30496-0

**Published:** 2022-06-15

**Authors:** Mrinal M. Gounder, Narasimhan P. Agaram, Sally E. Trabucco, Victoria Robinson, Richard A. Ferraro, Sherri Z. Millis, Anita Krishnan, Jessica Lee, Steven Attia, Wassim Abida, Alexander Drilon, Ping Chi, Sandra P. D’ Angelo, Mark A. Dickson, Mary Lou Keohan, Ciara M. Kelly, Mark Agulnik, Sant P. Chawla, Edwin Choy, Rashmi Chugh, Christian F. Meyer, Parvathi A. Myer, Jessica L. Moore, Ross A. Okimoto, Raphael E. Pollock, Vinod Ravi, Arun S. Singh, Neeta Somaiah, Andrew J. Wagner, John H. Healey, Garrett M. Frampton, Jeffrey M. Venstrom, Jeffrey S. Ross, Marc Ladanyi, Samuel Singer, Murray F. Brennan, Gary K. Schwartz, Alexander J. Lazar, David M. Thomas, Robert G. Maki, William D. Tap, Siraj M. Ali, Dexter X. Jin

**Affiliations:** 1 Memorial Sloan Kettering Cancer Center, New York, NY USA; 2 Weill Cornell Medical College, New York, NY USA; 3 Foundation Medicine, Inc., Cambridge, MA USA; 4 Mayo Clinic, Jacksonville, FL USA; 5 City of Hope, Duarte, CA USA; 6 Sarcoma Center of Santa Monica, Santa Monica, CA USA; 7 Massachusetts General Hospital, Cambridge, MA USA; 8 Harvard Medical School, Boston, MA USA; 9 University of Michigan, Ann Arbor, MI USA; 10 Johns Hopkins Sidney Kimmel Comprehensive Center, Baltimore, MD USA; 11 Montefiore Medical Center, Albert Einstein College of Medicine, Bronx, NY USA; 12 University of California at San Francisco, San Francisco, CA USA; 13 James Cancer Center, Ohio State University, Columbus, OH USA; 14 The University of Texas MD Anderson Cancer Center, Houston, TX USA; 15 University of California at Los Angeles, Los Angeles, CA USA; 16 Dana-Farber Cancer Institute, Boston, MA USA; 17 Albany Medical College, Albany, NY USA; 18 Herbert Irving Cancer Center, Columbia University, New York, NY USA; 19 Garvan Institute of Medical Research, Darlinghurst,, NSW Australia; 20 Abramson Cancer Center, University of Pennsylvania, Philadelphia, PA USA

**Keywords:** Targeted therapies, Cancer genomics, Sarcoma, Tumour heterogeneity, Tumour biomarkers

## Abstract

There are more than 70 distinct sarcomas, and this diversity complicates the development of precision-based therapeutics for these cancers. Prospective comprehensive genomic profiling could overcome this challenge by providing insight into sarcomas’ molecular drivers. Through targeted panel sequencing of 7494 sarcomas representing 44 histologies, we identify highly recurrent and type-specific alterations that aid in diagnosis and treatment decisions. Sequencing could lead to refinement or reassignment of 10.5% of diagnoses. Nearly one-third of patients (31.7%) harbor potentially actionable alterations, including a significant proportion (2.6%) with kinase gene rearrangements; 3.9% have a tumor mutational burden ≥10 mut/Mb. We describe low frequencies of microsatellite instability (<0.3%) and a high degree of genome-wide loss of heterozygosity (15%) across sarcomas, which are not readily explained by homologous recombination deficiency (observed in 2.5% of cases). In a clinically annotated subset of 118 patients, we validate actionable genetic events as therapeutic targets. Collectively, our findings reveal the genetic landscape of human sarcomas, which may inform future development of therapeutics and improve clinical outcomes for patients with these rare cancers.

## Abstract (web summary)

Comprehensive molecular profiles are required to understand and treat sarcomas, which comprise more than 70 different subtypes. Here, the authors profile the genomic landscape of 7494 sarcomas across 44 histologies using targeted panel sequencing and identify potential therapeutic targets.

## Introduction

The term “sarcoma” is shorthand for a complex family of more than 70 different diseases arising from connective tissue, independent of anatomic location, with each histology having unique natural history, biology, genetics, prognosis, and treatment^1–4^. These rare cancers comprise 1–2% of adult cancers worldwide, representing 6–15% of childhood cancer (<15 years) and 11% of adolescent and young adult cancers (15–29 years); the estimated annual incidence in the United States is 15,000 patients^5,6^. Due to their rarity and heterogeneity, sarcomas present particular challenges for accurate diagnosis, prognosis, and treatment. For instance, diagnostic errors in sarcoma remain exceedingly common, with rates up to 25% even among expert sarcoma pathologists^7–9^.

Most localized sarcomas are treated by en bloc surgical resection with or without radiation. With few exceptions (gastrointestinal stromal tumors [GISTs], Ewing sarcoma, rhabdomyosarcoma, osteosarcoma), the benefit of adjuvant therapies remains controversial^2–4,10^. Systemic therapies are palliative in the metastatic setting, where median overall survival is 11–20 months^2–4^. Few molecular-guided therapeutics have proven efficacy for sarcomas; these are limited to imatinib and others for *KIT-* or *PDGFRA-*mutated GIST and *COL1A1(A3)-PDGFA(D)* fusion-driven dermatofibrosarcoma protuberans (DFSP), and multi-tyrosine kinase inhibitors for *ALK* and *NTRK* fusion-driven inflammatory myofibroblastic tumors (IMT) and sarcoma NOS^11–14^.

The lack of effective targeted therapies for most sarcomas may be partially addressed by augmenting the limited available knowledge of the mutational landscape of mesenchymal tumors, which are much less studied compared with epithelial and neural-derived cancers. To date, genomic studies in sarcoma, such as those coordinated through The Cancer Genome Atlas (TCGA), have been limited by small size, a focus on early-stage disease, few histologies (e.g., liposarcoma, leiomyosarcoma, osteosarcoma), and scant clinical outcomes data^15–27^.

In this work, we illustrate the genomic landscape in 7494 patients spanning 44 distinct sarcoma subtypes, revealing the potential clinical utility of targeted next-generation sequencing in diagnosis, prognosis, and management of connective tissue malignancies.

## Results

### Patient cohort

**Fig. 1:**
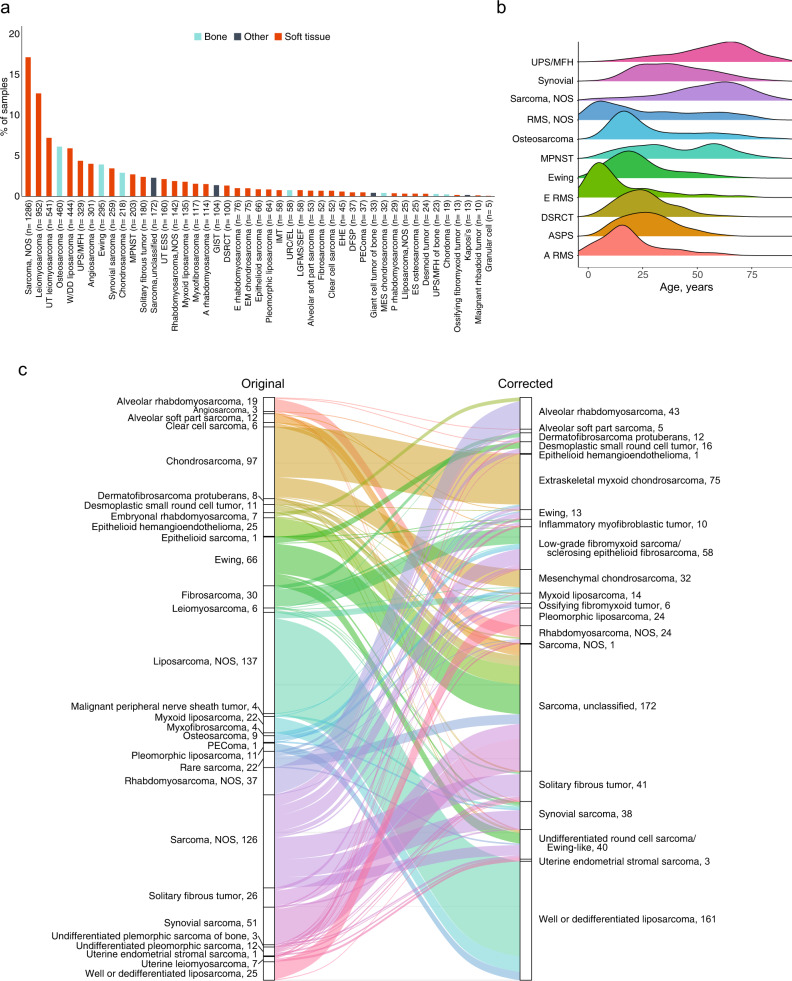
Cohort characterization and diagnostic corrections of sarcoma subtypes. **a** Distribution of soft tissue and bone sarcoma subtypes among the cohort of 7494 patients. **b** Density curves showing age distributions for common pediatric, adolescent, and young adult (P-AYA; defined as ≤30 years of age) cancers. Vertical lines represent median age for each sarcoma. **c** Sankey diagram illustrating diagnostic corrections for 10.5% (789/7,494) of patient samples. Left, number of cases identified for each subtype according to original submitted pathology results; right, number of corrections or refinements for each histology as determined by the presence or absence of pathognomonic genomic rearrangements or signatures. NOS not otherwise specified, W/DD well or dedifferentiated, UPS undifferentiated pleomorphic sarcoma, MFH malignant fibrous histiocytoma, MPNST malignant peripheral nerve sheath tumor, UT ESS uterine endometrial stromal sarcoma, A alveolar, GIST gastrointestinal stromal tumor, DSRCT desmoplastic small round cell tumor, E embryonal, EM extraskeletal myxoid, IMT inflammatory myofibroblastic tumor, URC/EL undifferentiated round cell/Ewing-like, LGFMS/SEF low-grade fibromyxoid sarcoma/sclerosing epithelioid fibrosarcoma, EHE epithelioid hemangioendothelioma, DFSP dermatofibrosarcoma protuberans, PEComa perivascular epithelioid cell tumor, MES mesenchymal, P pleomorphic, ES extraskeletal, RMS rhabdomyosarcoma. Source data are provided as a Source Data file.

From 2012 to 2018, 7494 patients diagnosed with sarcoma consented to tumor profiling to help inform clinical management of their disease. Tumor tissue (without normal tissue) was profiled by massively parallel, next-generation sequencing (NGS) of 465 genes, select introns of 31 genes involved in rearrangements, and RNA sequencing (cDNA) of 333 commonly rearranged genes to better identify de novo and rare gene fusions^28^ using the FoundationOne HEME^TM^ platform (Supplementary Table S1). Soft tissue, bone, and “other” sarcomas represented 81.0% (*n* = 6067), 14.7% (*n* = 1105), and 4.3% (322) of the sequenced tumors, respectively, with the most common types being sarcoma, not otherwise specified (NOS) (17.2%) and leiomyosarcoma (LMS) (12.7%) (Fig. 1a; individual sample data in Supplementary Data 1). Based on well-established criteria, we broadly categorized sarcomas as either translocation-associated (*n* = 1724, 23.0%) or genomically complex and other which either display multiple, complex karyotypes with no specific patterns or harbor specific, recurring alterations (*n* = 5770, 77.0%)^1,2,29^ (Supplementary Table S2). Patients’ median age was 53 years (range <1–89 years) and 53.4% were female. Pediatric, adolescent, and young adult (P-AYA) patients, defined as age ≤30 years, constituted 21.8% (1636/7494) of the cohort. Age distribution varied among sarcoma types (Fig. 1b). The mean computational tumor purity was 56.5% and specimens were sequenced to a median depth of 704X (interquartile range [IQR] 515–798X). An average of 3.8 known or likely pathogenic genomic alterations were identified per patient. A total of 28,546 known or likely pathogenic variants (11,536 non-synonymous single nucleotide variants [SNVs]/indels, 13,239 copy number alterations, and 3771 rearrangements) were detected (Supplementary Fig. S1; individual variant calls in Supplementary Data 1). No known or likely pathogenic alterations were detected in 226 (3.0%) samples using this gene panel. Variants of unknown significance were excluded from analyses.

### Diagnostic refinements

Based on sequencing results, we estimated that 789 (10.5%) of patients had a potentially incorrect diagnosis and/or might benefit from further diagnostic assessment (Fig. 1c and Supplementary Table S3). Re-classifications mostly resulted from the detection of highly histology-specific and recurrent pathognomonic translocations or alterations. For example, an initial diagnosis of “sarcoma NOS” was re-classified as synovial sarcoma (24 cases) when the *SS18-SSX1/2/4* gene fusions were identified. We determined that 8.9% (126/1411) of cases initially diagnosed as sarcoma NOS could be reclassified to a specific sarcoma subtype. Sarcomas lacking expected pathognomonic fusions (for example, lack of *NAB2-STAT6* in a solitary fibrous tumor) were noted as potential diagnostic errors and termed “sarcoma, unclassified” (*n* = 172), while a non-specific diagnosis of liposarcoma was further refined to well/dedifferentiated liposarcoma when *MDM2* gene amplification was found. Genomic alterations that could further specify or possibly change diagnosis were most common among patients whose subtype assignment in the database was sarcoma NOS; liposarcoma NOS; chondrosarcoma; Ewing sarcoma; and synovial sarcoma. Diagnoses were only reclassified when there was a high degree of confidence that the molecular findings (presence or absence of pathognomonic alterations) alone warranted a change in pathological diagnosis based on current knowledge^1^. However, these potential diagnostic errors and subsequent reclassifications were not reconfirmed by expert pathology review. Therefore, tumors that were termed “sarcoma, unclassified” were not categorized into any specific sarcoma subtypes and excluded from disease-specific analysis.

### Base substitutions, indels (short variants), and copy number variations

**Fig. 2:**
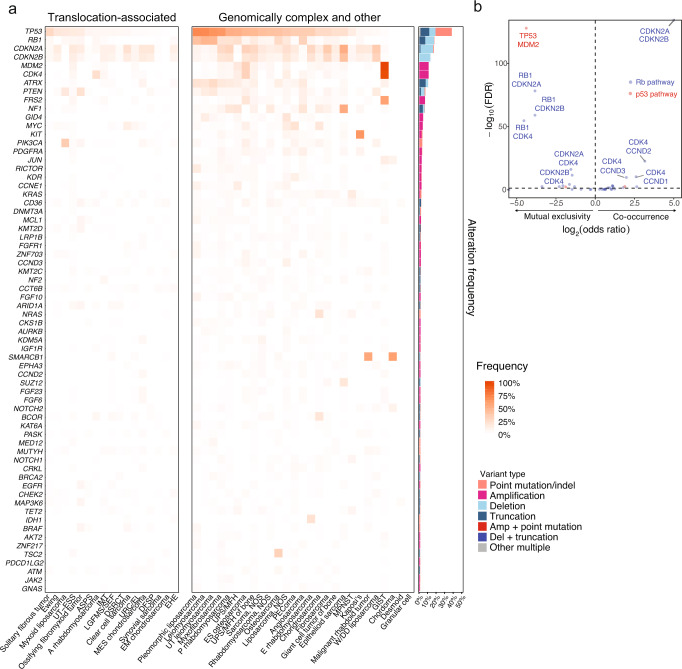
Genomic landscape of sarcomas. **a** Heat map showing the frequency of recurrent short variants and copy number variants in the listed genes in each subtype of sarcoma, grouped as translocation-associated or genomically complex and other sarcomas (left), as well as the types of genomic alterations (right). **b** Volcano plot showing co-occurrence (odds ratio >1) and mutual exclusivity (odds ratio <1) between two genes within the same pathway. Top ten significant interactions (FDR < 0.05) are labeled. UT ESS uterine endometrial stromal sarcoma, ASPS alveolar soft part sarcoma, A alveolar, IMT inflammatory myofibroblastic tumor, LGFMS/SEF low-grade fibromyxoid sarcoma/sclerosing epithelioid fibrosarcoma, DSRCT desmoplastic small round cell tumor, URC/EL undifferentiated round cell/Ewing-like, DFSP dermatofibrosarcoma protuberans, EHE epithelioid hemangioendothelioma, UT uterine, P pleomorphic, UPS undifferentiated pleomorphic sarcoma, MFH malignant fibrous histiocytoma, ES extraskeletal, NOS not otherwise specified, PEComa perivascular epithelioid cell tumor, E embryonal, MPNST malignant peripheral nerve sheath tumor, W/DD well or dedifferentiated, GIST gastrointestinal stromal tumor. Source data are provided as a Source Data file.

The highest frequencies of short and copy number variants were seen in key regulators of genomic stability such as *TP53* (37.1%) and *MDM2* (10.7%), and cell cycle regulators including *RB1* (17.2%), *CDKN2A* (16.7%), *CDKN2B* (12.8%), *CDK4* (10.6%), and *CDK6* (0.7%) (Fig. 2a, genes with alteration frequencies of ≥1%; Supplementary Fig. S2, genes with observed alteration frequencies of <1% in the total sarcoma cohort and ≥5% in any individual sarcoma).These classes of alterations were also seen in *MDM4* (0.5%) and related cell-cycle genes *CCNE1* (2.6%), *CCND1* (1.0%), *CCND2* (1.4%), and *CCND3* (1.8%). Short variants accounted for the majority of *TP53* alterations (79.3%), whereas *CDKN2A* and *CDKN2B* alterations were mostly homozygous copy number deletions (84.5% and 99.0%, respectively) and *MDM2*, *CDK4/6, CCND1/2/3*, and *CCNE1* were typically amplified (>94% of all alterations for each gene).

The p53 tumor suppressor pathway (*TP53, MDM2, MDM4*) and Rb pathway, including associated canonical cell cycle genes (*RB1, CDKN2A/B, CDK4/6, CCND1/2/3, CCNE1*), were altered in 47.8% and 46.8% of all sarcomas, respectively^30–32^. Alterations in the p53 and Rb pathways strongly co-occurred following FDR adjustment (OR = 4.2, *p* < 1.3 × 10^−196^) (Fig. 2b, Supplementary Table S4). However, alterations within each pathway, i.e. *TP53 vs. MDM2/4* and *RB1* vs. *CDKN2A/B* vs. *CDK4/6*, were mutually exclusive after FDR adjustment. An interesting exception was *CCND1/2/3* (but not *CCNE1*), in which alterations were strongly co-occurrent with those in *CDK4/6* and *CDKN2A/B*.

The frequencies of alterations differed between complex sarcomas and translocation-associated sarcomas within *TP53* (44.9% vs. 11.3%)*, RB1* (21.5% vs 2.8%), *CDKN2A* (19.0% vs. 9.0%), *MDM2* (13.5% vs. 1.6%), *CDK4* (12.8% vs. 3.3%), *MDM4* (0.6% vs. 0.2%), *CCND1* (1.2% vs. 0.3%), *CCND2* (1.7% vs. 0.3%), *CCND3* (2.3% vs. 0.2%), and *CCNE1* (3.2% vs. 0.5%). Together, compared with translocation-associated sarcomas, genomically complex sarcomas harbored higher frequencies of p53 pathway (58.2% vs. 12.9%) and Rb pathway (56% vs. 15.8%) alterations.

Oncogenic drivers common in melanoma or epithelial cancers, such as *BRAF* V600E (0.5%) *KRAS* G12X (0.9%), and exon 19 deletions (0.01%) and L858R (0.01%) mutations in *EGFR*, are rare in mesenchymal sarcomas and raise the possibility of identifying epithelial malignancies with sarcomatoid differentiation. Genes in which alterations were enriched in specific sarcomas included those from the 4q12 amplicon (*KDR, PDGFRA, KIT*) in osteosarcoma (10.7%), liposarcoma NOS (8.0%), and UPS/MFH (7.3%); from the 11q13 amplicon (*CCND1, FGF2, FGF3, FGF19*) in UPS of bone (4.3%); *CDKN2A/B* deletion in malignant peripheral nerve sheath tumors (MPNST; 47.3%) and chordoma (36.8%); *SMARCB1* in epithelioid sarcoma and malignant rhabdoid tumor (56% and 60%, respectively); *PIK3CA, PTEN*, and *AKT* (specifically E17K mutations) in myxoid liposarcoma (36.3%, 17.8%, and 3.7%, respectively); *IDH1* in chondrosarcoma (22.9%); mutations in *PDCD11*^*V334I*^ and amplifications in *HRAS* in granular cell tumor (20% each); *PBRM1* inactivating alterations in chordoma (15.8%); amplifications in *PRSS8* in low-grade fibromyxoid sarcoma/sclerosing epithelioid fibrosarcoma (6.9%); and *C-MYC* amplification in embryonal rhabdomyosarcoma (18.4%), clear cell sarcoma (13.5%), and osteosarcoma (15.0%).

### Translocations and kinase fusions

**Fig. 3:**
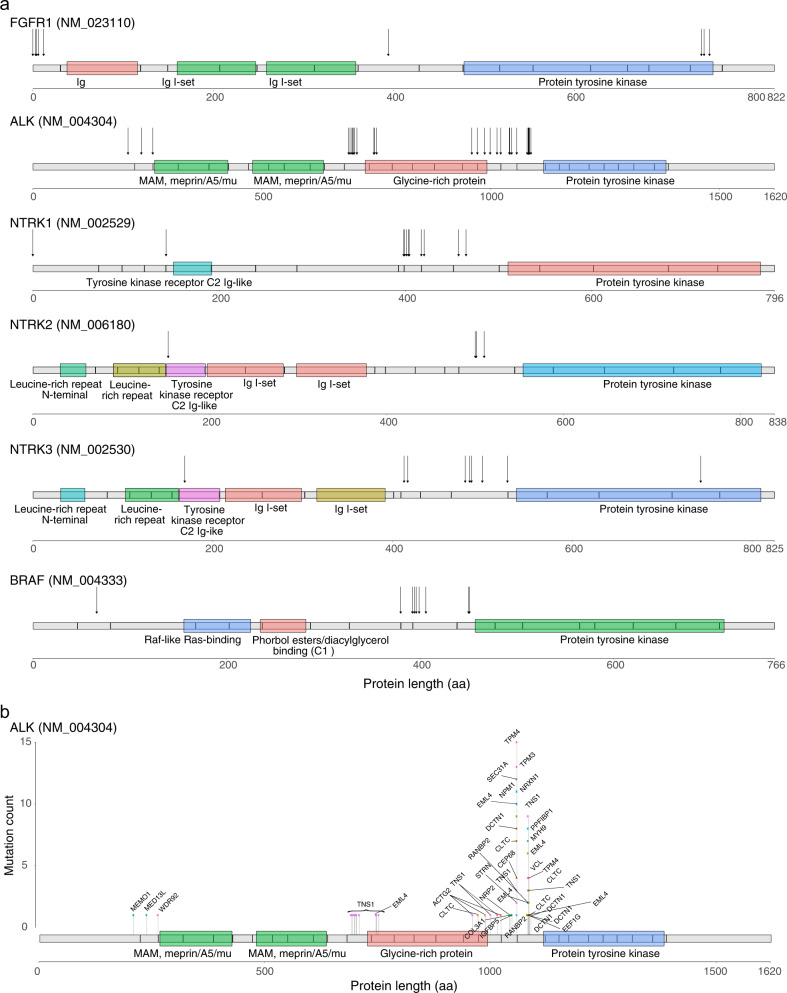
Gain-of-function kinase gene fusions. **a** Recurrent kinase gene fusions resulting in activating oncogenes in the kinase domains of FGFR1, ALK, NTRK1, NTRK2, NTRK3, and BRAF. Arrows indicate breakpoints. **b** Rearrangements that result in activating fusions between TNS1 and ALK, including breakpoints in introns 18 and 16 that give rise to fusions of exons 19–29 and 17–29, respectively. These fusions were detected in 16 patients primarily in uterine and non-uterine leiomyosarcoma. Source data are provided as a Source Data file.

Actionable kinase fusions in sarcoma have not been comprehensively described with the exception of *ALK* fusions in inflammatory myofibroblastic tumors (IMT) and *NTRK1–3* fusions in infantile fibrosarcoma^33–35^. Gain-of-function fusions involving kinase genes represent potentially druggable targets^36,37^. We identified potentially actionable kinase fusions in 2.6% (196/7494) of all sarcomas. These included *ALK, BRAF, FGFR1–4, NTRK1–3, RET*, and *ROS1* kinase fusions in a wide range of sarcoma histologies, including IMT (62.1%), MPNST (4.9%), UPS of bone (4.3%), extraskeletal osteosarcomas (4%), UPS (3.6%), sarcoma NOS (3.8%), and LMS (1.9%) (Fig. 3a). Interestingly, multiple breakpoints were identified for *ALK* fusions at exon 12 near the glycine-rich domain (Fig. 3b). An emerging fusion partner of *ALK*, *TNS1*, was recently described in uterine leiomyosarcomas; we observed this fusion in 16 cases (12 cases in uterine and non-uterine LMS) in the current cohort. These results suggest that *TNS1-ALK* rearrangements are a recurrent fusion in LMS and may require further classification^38,39^.

Overall, 42.5% (*n* = 3182) of all sarcomas had structural rearrangements, including some well-known and presumed activating fusions of transcription factors. A frequently fused gene was *EWSR1*, for which we identified well-known (*FLI1, WT1, NR4A3)* and lesser known partners that presumably also function as transcriptional regulators^21,40^ (Supplementary Fig. S3a). We also observed rearrangements involving genes such as *RB1* (1.5%), *TP53* (1.4%), *LRP1B* (0.6%), *ATRX* (0.5%), *ARID2* (0.2%), *BRCA2* (0.3%), and *NOTCH3* (0.3%), but the functional significance of these rearrangements remains untested (Supplementary Fig. S3b).

### Alterations in DNA damage repair, tumor mutational burden, and genomic signatures

Immune checkpoint inhibitors have demonstrated clinical benefit in select sarcoma subtypes^41–43^. There is limited knowledge of the landscape of potential genomic biomarkers of efficacy of these agents such as mismatch or homologous recombination deficiency, microsatellite instability (MSI-H), or tumor mutational burden (TMB) in sarcomas^44,45^. Mismatch repair deficiency (MMR-D, defined as loss or inactivation of *MLH1, MSH2, MSH3, MSH6, PMS2*) was found in 2.1% of all sarcomas; these alterations were mono- or biallelic (Supplementary Fig. S4a). The median TMB in MMR-D tumors was 6.5 mut/Mb (IQR 1.8–17.8) and significantly higher than that in MMR-proficient tumors (2.4 mut/Mb; IQR 0.8–4.0; *p* < 0.01). Microsatellite instability (MSI-High, evaluable in 6206 cases) was observed in only 18 cases (0.29%; most frequently uterine endometrial stromal sarcoma, leiomyosarcoma, and sarcoma NOS); with a median TMB of 25.8 mut/Mb (IQR 19.8–30.1); 14 of these patients had corresponding alterations in mismatch repair genes.

**Fig. 4:**
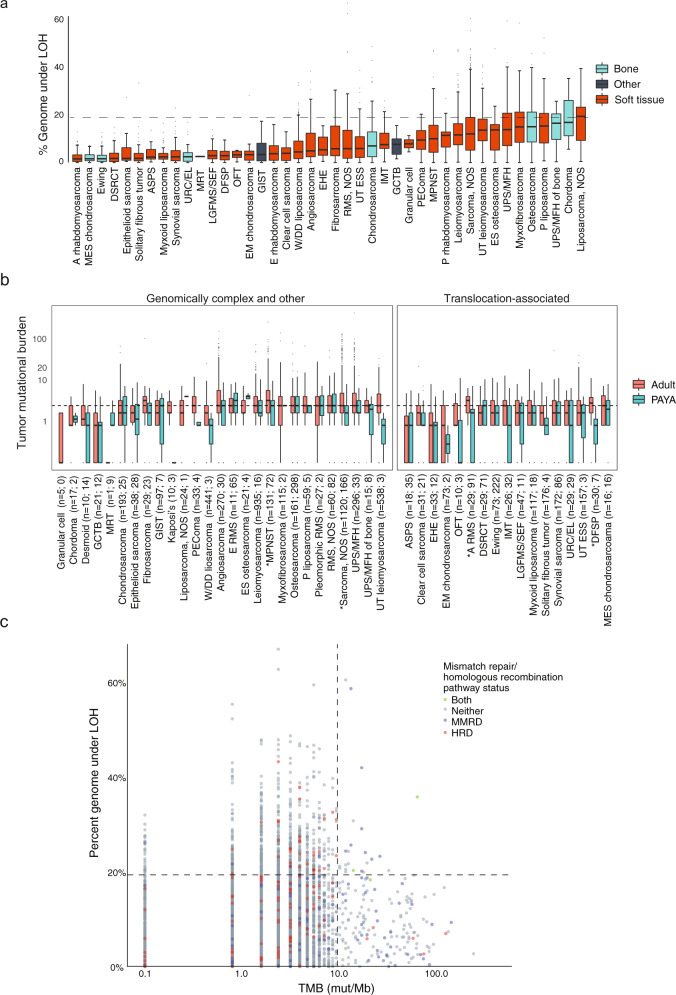
Mutational burden and genomic loss of heterozygosity. **a** Box-and-whisker plot of genomic loss of heterozygosity (gLOH) expressed as % of genome under LOH for each sarcoma histology. gLOH only evaluable *n* = 4619. Dashed horizontal line (19.3%) indicates 1 standard deviation above the mean gLOH. **b** Box-and-whisker plot of tumor mutational burden (TMB) and signatures derived from sequencing data for each sarcoma histology, grouped by age: pediatric, adolescent, and young adult (P-AYA) versus adult (>30 years). In (**a**, **b**), the lower and upper box boundaries represent 25th and 75th percentiles, lines within boxes represent medians, whiskers extend to extreme values ≤1.5 x IQR, and points beyond whiskers are outliers. Asterisk (*) indicates significant difference (with an FDR < 0.05) using a two-tailed non-parametric Mann–Whitney *U* test. In all cases, P-AYA harbored significantly lower TMB. **c** Relationship between tumor mutational burden (TMB) and genomic loss of heterozygosity (gLOH). Vertical line (10 mut/Mb) indicates distinction between “low” and “high” TMB. Dashed horizontal line (19.3%) indicates 1 standard deviation above the mean gLOH and indicates distinction between “low” and “high” gLOH. A alveolar, MES mesenchymal, DSRCT desmoplastic small round cell tumor, ASPS alveolar soft part sarcoma, URC/EL undifferentiated round cell/Ewing-like, LGFMS/SEF low-grade fibromyxoid sarcoma/ sclerosing epithelioid fibrosarcoma, DFSP dermatofibrosarcoma protuberans, OFT ossifying fibromyxoid tumor, EM extraskeletal myxoid, GIST gastrointestinal stromal tumor, E embryonal, W/DD well/dedifferentiated, EHE epithelioid hemangioendothelioma, RMS rhabdomyosarcoma, NOS not otherwise specified, UT uterine, ESS endometrial stromal sarcoma, IMT inflammatory myofibroblastic tumor, GCTB giant cell tumor of bone, PEComa perivascular epithelioid cell tumor, MPNST malignant peripheral nerve sheath tumor, P pleomorphic, ES extraskeletal, UPS undifferentiated pleomorphic sarcoma, MFH malignant fibrous histiocytoma. Source data are provided as a Source Data file.

Overall, 2.5% (184/7494) of samples harbored pathogenic alterations in homologous recombination repair pathways (*BRCA1/2, PALB2, RAD51*, and its paralogs *RAD51B, RAD51D, RAD52, RAD54L*). Supplementary Figure S4b. Among these, 72 were confirmed biallelic losses, which were most common in uterine leiomyosarcoma (4.8%, 26/541), angiosarcoma (2.0%, 6/301), and leiomyosarcoma (1.6%, 15/952). To confirm whether this results in a homologous recombination deficiency (HRD) phenotype or genomic “scars”, we evaluated genomic loss of heterozygosity (gLOH), defined as the number of regions affected by LOH, excluding whole chromosome or chromosome arm losses^46^ in a subset of patients (61.6%, 4619/7494) where gLOH could be determined and samples had passed a copy-number based quality control metric of signal to noise ratio. The median gLOH across all sarcomas was 8.6% (range 0–66.8%, IQR 3.0–15.7%) (Fig. 4a), which was significantly lower than the median gLOH of those with mono- or biallelic HRD alterations (14.4%, IQR 5.9–20.3%; *p* < 2 × 10^−6^) and of those with only biallelic HRD alterations (17.9%; IQR: 14.5–24.2; *p* < 4 × 10^−12^). We set an arbitrary threshold of 1 standard deviation above the mean as indicative of “high” gLOH, which corresponded to a cutoff of ≥19.3%. In our cohort, 15% (697/4619) of all sarcomas showed high gLOH, of which only a fraction was explained by alterations in HRD genes (5.0%, 35/697) and even fewer by biallelic HRD alterations (3.3%, 23/697). This is exemplified in liposarcoma NOS, in which 50.0% had high gLOH but only 4.0% had alterations in the HRD gene set, with similar patterns in osteosarcoma (30.6% vs. 1.7%) and UPS (26.6% vs. 3.6%). The mechanism of gLOH in a majority of sarcomas remains to be further investigated.

Across sarcomas, the median TMB was 2.4 mut/Mb (IQR 0.8–4.0) and TMBs of ≥5, ≥10 and ≥15 mut/Mb were found in 13.0%, 3.9%, and 2.9%, respectively (Fig. 4b). Translocation-associated sarcomas had lower TMB than genomically complex sarcomas, with a median of 1.6 vs. 2.4 mut/Mb; *p* < 4 × 10^−61^. TMB ≥ 10 mut/Mb was most frequent among angiosarcoma (15.0%), UPS (10.9%), MPNST (10.8%), and ossifying fibromyxoid tumor (7.7%). Importantly, gLOH and high TMB were non-overlapping, suggesting distinct biological processes (Fig. 4c).

Lastly, the *CD274* gene, which encodes the PD-L1 protein, was amplified in 1.0% (75/7594) of all sarcomas, with the highest rates of amplification in UPS/MFH (3.6%), myxofibrosarcoma (2.6%) and sarcoma NOS (2.2%). *CD274*-amplified tumors had a median TMB of 3.2 mut/Mb. We were unable to correlate our findings with PD-L1 immunohistochemistry; *CD274* amplification warrants prospective evaluation in sarcomas^47,48^.

**Fig. 5:**
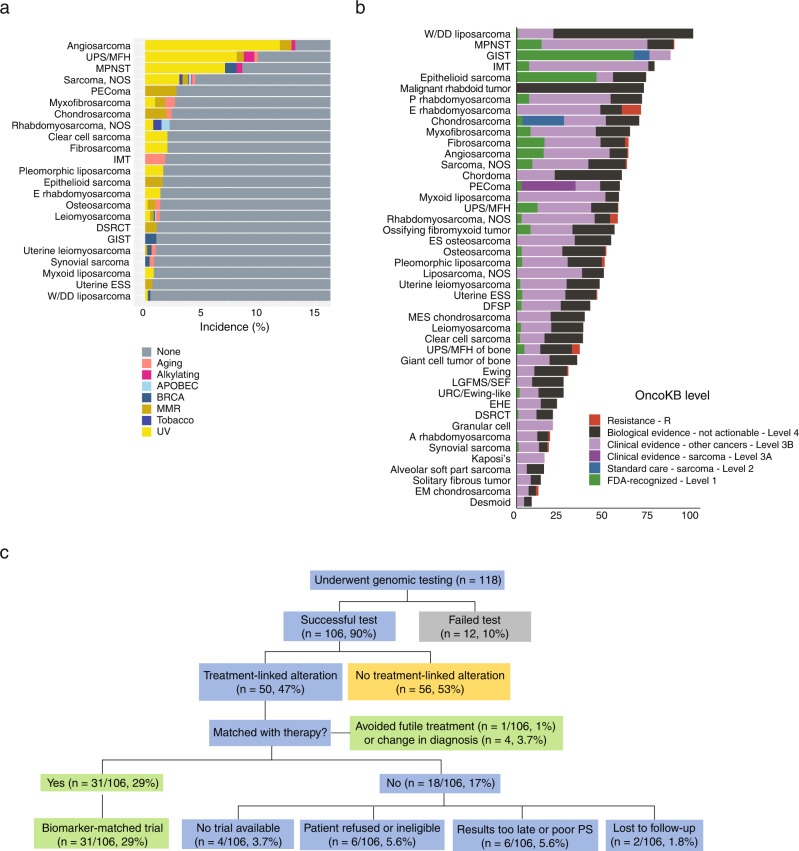
Actionability. **a** Dominant mutational signatures in various sarcoma histologies. **b** Evidence for clinical actionability of somatic alterations across 44 sarcoma histologies. SOC, standard of care. **c** Flow chart of actions taken as a result of comprehensive genomic sequencing in 118 patients at MSK who underwent Foundation Medicine sequencing. OncoKB classifications were not applied retrospectively for the MSK cohort and thus reflect prevailing knowledge at time of enrollment. APOBEC, apolipoprotein B mRNA editing catalytic polypeptide-like; MMR, mismatch repair deficiency. Tobacco signifies mutational genomic signature attributable to cancers derived from tissues potentially exposed to tobacco smoke. UPS undifferentiated pleomorphic sarcoma, MFH malignant fibrous histiocytoma, MPNST malignant peripheral nerve sheath tumor, NOS not otherwise specified, PEComa perivascular epithelioid cell tumor, IMT inflammatory myofibroblastic tumor, E embryonal, DSRCT desmoplastic small round cell tumor, GIST gastrointestinal stromal tumor, ESS endometrial stromal sarcoma, W/DD well or dedifferentiated, P pleomorphic, ES extraskeletal, DFSP dermatofibrosarcoma protuberans, MES mesenchymal, LGFMS/SEF low-grade fibromyxoid sarcoma/sclerosing epithelioid fibrosarcoma, URC undifferentiated round cell, EHE epithelioid hemangioendothelioma, A alveolar, EM extraskeletal myxoid. Source data are provided as a Source Data file.

Other origins of elevated TMB can be inferred from mutational signatures, including that of ultraviolet light (UV)-induced damage^49^. Assessment of mutational signatures revealed that the UV signature was dominant in cutaneous angiosarcoma (35/41 angiosarcoma samples in which mutational signatures were evaluable; 22 of these arose in the skin), as well as in UPS and MPNST (Fig. 5a). Strikingly, in the ultrarare (11, 0.15%) cases that harbored very high TMB (≥100 mut/Mb), a UV signature was usually present (77.8%, 7/9 evaluable), which suggests either an anatomic origin with exposure to solar radiation or sampling of a metastatic site with UV exposure. Interestingly, these cases also harbored frequent *NF1* mutations (45.5%, 5/11) and thus, the co-occurrence of *NF1* alterations and a UV signature may suggest an alternate diagnosis of desmoplastic melanoma (S100+, melanA/HMB45-negative) or radiation-associated MPNST^50^.

### Differences between pediatric, adolescent and young adult (P-AYA), and adult patients

In childhood sarcomas such as rhabdomyosarcoma, osteosarcoma, and Ewing, patient age has emerged as an independent prognostic factor that influences overall survival; older patients fare worse^51–54^. We evaluated whether genomic differences could contribute to the observed differences in survival. We did not detect any significant differences in genomic alterations using an age cutoff of ≤30 years in any sarcoma subtype with two exceptions: sarcoma NOS and osteosarcoma. In osteosarcoma, copy number variation (CNV) differed between P-AYA (*n* = 298) and adult (*n* = 161) patients. In particular, CNV was more frequent in *CCND3, AURKB, CCNE1, GID4*, and *MYC* in P-AYA, while *MDM2, CDKN2A/B*, and *FRS2* were more frequently altered in adult osteosarcoma (*p* < 0.001) (Supplementary Fig. S5).

Recently, high TMB has emerged as a negative prognostic factor in rhabdomyosarcoma^55^. As TMB increases with age^56^, we compared TMB within each sarcoma subtype between P-AYA and adult patients (Fig. 4b). We confirmed that both median TMB and the proportion of patients with high TMB (≥10 MB), regardless of TMB cutoff, were higher among adult patients. Within each sarcoma, TMB differed significantly between P-AYA and adults in sarcoma NOS (1.6 vs. 2.4 mut/Mb; FDR < 7 × 10^−11^), alveolar rhabdomyosarcoma (1.6 vs. 3.2 mut/Mb; FDR < 0.0013), MPNST (2.4 vs. 3.2 mut/Mb; FDR < 0.004), and DFSP (0.8 vs. 2.8 mut/Mb; FDR < 0.05). TMB remained significantly lower among P-AYA compared with older adult patients even within translocation-associated (*p* < 0.0008) and complex sarcomas (*p* < 3 × 10^−14^). The clinical prognostic implications of this difference in TMB are not clear.

Compared with adults, P-AYA patients typically had lower median gLOH (“genomic scars”) at 3.8% vs. 9.5% (*p* < 4 × 10^−43^); the proportion of cases with high gLOH was also lower among P-AYA patients at 16.0% vs. 11.5% (*p* < 5 × 10^−4^). When analyzing within each sarcoma subtype, gLOH was often significantly lower in P-AYA relative to adults in sarcoma NOS (4.8% vs. 12.5%; FDR < 9 × 10^−8^), undifferentiated round cell/Ewing-like sarcomas (0.5% vs. 3.0%; FDR < 0.009), rhabdomyosarcoma NOS (2.5% vs. 10.5%; FDR < 0.01), fibrosarcomas (3.2% vs. 14.4%; FDR < 0.03), and embryonal rhabdomyosarcomas (3.3% vs. 8.7%; FDR < 0.04). Again, an interesting outlier was osteosarcoma, where gLOH was significantly higher in P-AYA relative to adults (16.8% P-AYA vs. 12.2%; FDR < 0.001) (Supplementary Fig. S6).

### Clinical utility: actionable and resistance genes

To evaluate the potential impact of genomic profiling on selection of patients for treatment with FDA-approved or investigational drugs in clinical trials, we employed OncoKB (http://oncokb.org, data cutoff June, 8, 2021), a FDA-recognized, public, genetic variant database that provides information on the effects and treatment implications, including those for drug resistance, of genetic aberrations based on cancer type, FDA labeling, National Comprehensive Cancer Network (NCCN) guidelines, and scientific literature^57,58^. More than one-third of sarcoma patients (31.7%; *n* = 2372/7494) harbored at least one potentially actionable mutation (Fig. 5b), of which a minority (5.9%, *n* = 439) were FDA-recognized biomarkers for approved drugs in the given sarcoma type (OncoKB Level 1). Of note, GIST (*n* = 104, 1.4%) represented a small minority of our entire cohort. Alterations recognized as biomarkers for a specifically approved drug were, as expected, most frequent among GISTs, in which certain *KIT* mutations are an indication for treatment with imatinib; these were observed in 64.4% of GISTs. The majority of actionable mutations were Level 3B, denoting clinical benefit of the biomarker and drug in cancers other than sarcomas. Resistance mutations (primary and/or acquired) that could help avoid non-beneficial therapies were also observed and included *SDH* loss, *PDGFRA*^D842V^, and *KIT* mutations specifically associated with imatinib resistance in GIST, *ESR1* mutations potentially associated with anti-estrogen resistance in endometrial stromal sarcoma, inactivating *TP53* mutations associated with MDM2 inhibitor resistance, and *RB1* deletion associated with resistance to CDK4 inhibitors in dedifferentiated liposarcoma^59–65^. There were significantly higher observed frequencies (35.7 vs. 18.0%) of actionable mutations in genomically complex compared with translocation-associated sarcomas. Level 4 alterations (19.2%, *n* = 1442), while reported, are not considered actionable as these represent only preclinical evidence in sarcoma. Similarly, gLOH is not considered actionable.

### Clinical utility: patient characteristics and actionability

**Fig. 6:**
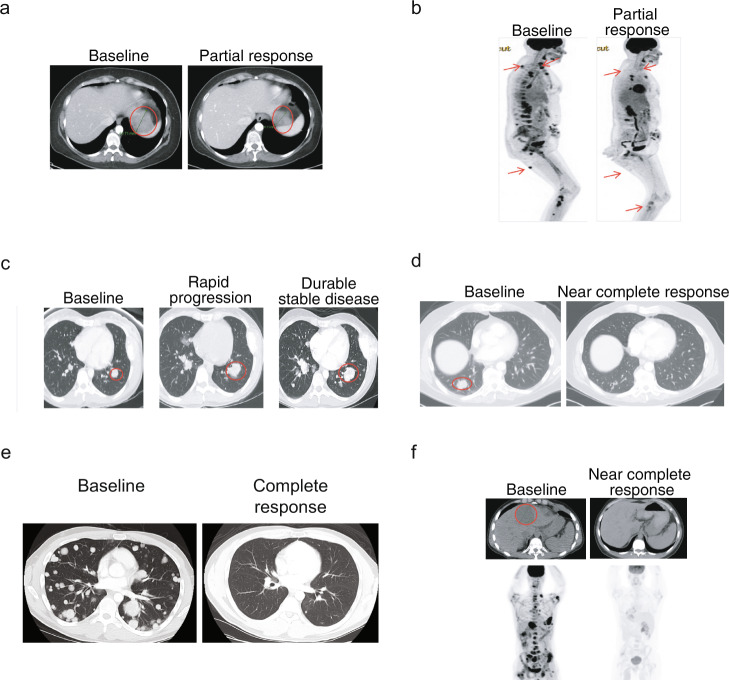
Treatment outcomes of selected patients in whom sequencing identified an actionable alteration. **a** Advanced, metastatic sarcoma, NOS harboring a *SMARCB1* deletion with durable partial response to tazemetostat, an EZH2 inhibitor. **b** Metastatic, refractory sarcoma harboring *BRAF*^V600E^ with a rapid and near complete response to vemurafenib and trametinib. **c** Metastatic osteosarcoma with rapid progression on doxorubicin and cisplatin. After sequencing, identification of an *ATM* exon 57-truncating mutation, a combination of a PARP inhibitor and an investigational drug led to durable stable of >1 year. **d** Near complete response in metastatic, refractory undifferentiated pleomorphic sarcoma with high TMB (20 mut/Mb) treated with pembrolizumab. **e** Complete response in refractory malignant PEComa (*TFE3* fusion-negative, *TSC1* subclonal) harboring intermediate tumor mutational burden (7 mut/Mb) treated with nivolumab and ipilumumab (compassionate, off-label). **f** Complete response in inflammatory myofibroblastic sarcoma harboring an *ETV6-NTRK3* fusion and treated with larotrectinib.

To begin to explore the potential impact of genomic profiling on patient outcomes, we analyzed clinical decisions taken based on FoundationOne genomic profiling as part of clinical management of patients treated at Memorial Sloan Kettering Cancer Center, NY (MSK). Pathology was reviewed at MSK. This cohort included 118 patients with 32 sarcoma histologies whose median age was 50 years (range 18–89); most had metastatic disease (60%) and had undergone a median of 2 (range 0–9) prior surgeries and received 4 (range 0–12) prior systemic therapies (Supplementary Table S5). In the MSK cohort, initial diagnosis by sarcoma pathologist was subsequently changed in 4% of patients based on results from genomic sequencing. In these cases, two patients with leiomyosarcoma were reclassified as dedifferentiated liposarcoma and therapy was changed to investigational MDM2 or CDK4 inhibitors, a third patient with sarcoma NOS was diagnosed as PEComa (*TSC2* loss) and recommended an mTOR inhibitor, and a fourth patient with MPNST was reclassified as synovial sarcoma based on an *SS18-SSX2* fusion and evaluated for NY-ESO-1-based T cell therapy. The median time from requiring systemic therapies to consenting for genomic profiling was 1.1 years (range 0–11.8), reflecting the use of genomic profiling later in the time course of managing refractory disease. At least one alteration was deemed actionable by the treating physician in 47% (*n* = 50) of patients, and 29% of the entire cohort was enrolled in a matched trial or off-label use of an FDA approved drug (Fig. 5c, Supplementary Table S6). Few radiographic responses are noted in Fig. 6. Two patients each with refractory, metastatic, sarcoma NOS harboring a *SMARCB1* deletion (Fig. 6a) and *BRAF*^V600E^ (Fig. 6b) had a durable partial response to tazemetostat, an EZH2 inhibitor and a rapid response to vemurafenib and trametinib, respectively. Another patient with metastatic osteosarcoma had rapid progression after doxorubicin and cisplatin. NGS identified an *ATM* exon 57-truncating mutation, an investigational combination of a PARP and ATR inhibitor led to durable stable disease of >1 year (Fig. 6c). Two patients each with metastatic, refractory undifferentiated pleomorphic sarcoma with high TMB (20 mut/mB) and advanced malignant PEComa with intermediate TMB (7 mut/Mb) had a near complete response (Fig. 6d) to pembrolizumab and nivolumab/ipilimumab, respectively (Fig. 6e). Lastly, a patient with inflammatory myofibroblastic sarcoma harboring a *ETV6-NTRK3* fusion had a durable, complete response to larotrectenib (Fig. 6f).

## Discussion

We report a cohort that comprises about 7500 patients with 44 sarcoma subtypes, including some common as well as many rare sarcomas that have not been genomically characterized. These data suggest that genomic profiling could be beneficial in the clinical management of some sarcoma patients, demonstrating its potential impact on precise diagnosis, prognosis, treatment decisions, and outcomes.

Based on sequencing results, we infer that the initial pathology diagnosis was potentially incorrect or could be refined in up to 10.5% of sarcoma patients. We acknowledge that these changes were not confirmed by an expert sarcoma pathologist; however, our findings are consistent with observations in our routine clinical practice and published studies^7,27,66–68^. We suspect that the diagnostic errors seen in our cohort of approximately 7500 patients is reflective of real-world data, as these patients received care worldwide at both academic and community hospitals, where access to sarcoma pathologists or specialized reagents including custom PCR or FISH probes may not be readily available. In the era of precision therapies, diagnostic errors can have profound implications for both prognosis and selection of treatments. Given the high costs and other logistical challenges, we do not suggest that all sarcoma patients undergo next generation sequencing. We also acknowledge that many sarcomas lack any specific gene signatures that can help pinpoint a specific subtype. However, given the complexity of soft tissue sarcomas and the lack of adequate diagnostic expertize in most centers globally, the integration of genomic profiling, where available, with morphologic, immunohistochemical, and cytogenetic (FISH) results along with the clinical context can assist the pathologist in diagnosis, particularly in challenging cases.

A third of sarcomas (31.7%, 2372/7494) in our cohort harbored at least one actionable alteration, i.e. one that may influence therapeutic decisions, with varying levels of supporting evidence^58^. Of note, GISTs, most of which carry actionable alterations, only constituted 1.4% (104/7494) of our entire cohort. Our finding is consistent with other published, albeit smaller, studies that have reported rates of actionable alterations ranging from 22–61% across sarcoma types^17,27,67^. This wide range reflects the variety of criteria applied to define a biomarker as actionable, and interpretation of these results remains challenging and controversial. We applied an FDA recognized, OncoKB criteria, which takes a strict view on actionability by considering the level of evidence for each biomarker and drug, as well as biological/cellular context^58^. Nevertheless, “actionability” is a dynamic term that will continuously change as our understanding of cancer biology and drug development evolves. Equally important, genomic sequencing may allow avoidance of harmful or non-beneficial therapies, exemplified by GIST harboring primary or acquired *SDH*^del^, *KIT*^EX13^, and *PDGFRA*^D842V^, known to indicate resistance to imatinib. Similarly, *ESR1* mutations in endometrial stromal sarcoma may predict primary or acquired resistance to hormonal therapy, as shown in breast cancer^69,70^. Lastly, although *RB1* and *TP53* mutations are uncommon in well/dedifferentiated liposarcoma, these alterations confer resistance to palbociclib (CDK4/6) and MDM2 inhibitors, respectively^71,65^. Although we defined actionability with respect to genomically matched targeted therapies, genomic findings can inform clinical action in other ways, including informing prognosis (e.g. *TP53, CDKN2A*, and *STAG2* in pediatric Ewing sarcoma)^21,72^.

Within the clinically annotated MSK cohort, genomic profiling informed selection of therapy in 29% (31/106) of patients and anecdotal benefits are described. OncoKB criteria were not applied retrospectively, and actionability categorizations herein reflected prevailing knowledge at the time of treatment. For example, one patient with an *NTRK* amplification and another with an *NTRK* fusion were enrolled in a clinical trial with a response noted in the patient with a fusion; this trial revealed that *NTRK* amplifications are not actionable^37^. Although a significant number of patients treated at MSK had actionable alterations, many were unable to receive corresponding therapies due to lack of access to or ineligibility for appropriate trials, loss to follow-up, or genomic profiling occurring late in the disease course. This is a sobering reminder that there is a wide gap, especially in rare cancers, between genomic findings and translational research impacting patient care. An example of this gap is the NCI-MATCH study, in which only 5% of patients with solid tumors were assigned to matched therapies at interim analysis (*n* = 645, including 20 patients with sarcomas)^73^. This highlights opportunities to improve equity in precision testing, increase research in rare cancers, broaden eligibility criteria, and improve access to clinical trials.

We show that kinase fusions (2.6%) are an important class of targetable oncogenic drivers across sarcomas^13,37,74–76^. The mechanism and functional significance of these fusions, as well as fusions involving *RB1, TP53, LRP1B, ATRX*, and others remain unknown and require further validation. The molecular mechanisms initiating and driving malignant transformation and metastases in sarcoma are poorly understood. Whole genome sequencing in a few common sarcomas (liposarcoma, leiomyosarcoma, osteosarcoma, Ewing sarcoma) has revealed a high incidence of alterations that affect the p53 and Rb pathways. Here, using a targeted gene panel, we show that co-alterations in tumor suppressor and cell cycle pathways are found in many, but not all, sarcomas. Some of the mechanisms involve inactivation of targets upstream of *TP53* (e.g. *MDM2*) and *RB1* (e.g. *CDK4*) that are consequently amenable to therapeutic strategies (e.g. MDM2 and CDK4 inhibitors) that reactivate wildtype p53 or arrest the cell cycle^77^. Our study did not interrogate other mechanisms such as chromosomal rearrangements, chromothripsis, epigenetic changes, loss of heterozygosity, and post-translational modifications that may further explain how mesenchymal cells acquire malignant potential. For example, *CDKN2A* homozygous deletions only explained loss of p16 in 50% of chordoma^78^. Similarly, in epithelioid sarcoma, while loss of INI-1 protein expression is near universal, the encoding *SMARCB1* gene is inactivated in only 56% of cases in our study (although these may also potentially be driven by intragenic copy number deletions), as previously reported with whole genome/exome sequencing^79^.

Ongoing trials of checkpoint inhibitors in sarcoma do not involve selection of patients with genomic biomarkers such as *CD274* (PD-L1) amplification or high MSI, TMB, or tumor-infiltrating lymphocytes, but instead are limited to histologic subtypes (e.g. LMS, UPS/MFH, ASPS) in which responses were seen in early clinical trials^41,42,47,80^. Here, we report the comprehensive landscape of TMB across sarcomas and note that 3.9% of patients harbor TMB ≥10 mut/Mb, which has therapeutic implications^81^. We acknowledge that several questions about high TMB in sarcoma remain unanswered. The FDA approval of pembrolizumab for cancers with TMB ≥10 mut/Mb based on the FoundationOne CDx assay was tissue-agnostic, but no sarcoma patients were enrolled in the KEYNOTE 158 study^82^. Anecdotally, one patient in our cohort with widely metastatic sarcoma was found to have a TMB of 7 mut/Mb and had a complete durable response with checkpoint blockade (Fig. 6g). While we acknowledge that whole exome sequencing (WES) is the gold standard for measuring TMB, panel-based assays provide reasonable estimations of TMB, and are now FDA approved diagnostic tests and are more convenient and affordable than WES for clinical use.

We also find that MSI-H is present in only 0.3% of sarcomas and therefore routine testing of all sarcoma specimens by IHC or PCR will have low yield. Our finding of a UV signature associated with high TMB in angiosarcoma was originally reported in our abstract and subsequently validated by other groups^83,84^. Further, the subtypes with high TMB—UPS, liposarcoma, angiosarcoma, osteosarcoma, chondrosarcoma, and uterine leiomyosarcoma—overlap with the list of subtypes for which clinical activity has been reported with checkpoint inhibitors^41,42^. Our data shows that genomic profiling, along with immunohistochemistry, may allow identification of predictive biomarkers of response to checkpoint inhibitors.

PARP inhibitors are being investigated as monotherapy or in combination with cytotoxic or checkpoint inhibitors for sarcomas due to emerging evidence of alterations in DNA damage repair pathways and/or HRD signatures in certain histologies^48,85–88^. There is also uncertainty as to whether *BRCA1/2* mutations are predictive of response to PARP inhibitors in settings other than ovarian, breast, prostate, and pancreatic cancers^89,90^. In our study, we examined HRD gene alterations, including those that were predicted to be biallelic; biallelic losses were most frequent in uterine leiomyosarcoma (4.8%) and chordoma (5.3%) and typically observed in <2% of most other sarcomas. We show that in the majority of samples, high gLOH scores are not necessarily explained by HRD gene alterations (5.0%). Other mechanisms such as aneuploidy likely contribute, as multiple complex pathways lead to the HRD phenotype. For example, in the Phase III ARIEL3 study, patients with *BRCA1/2* wildtype ovarian cancer that nonetheless harbored high gLOH (≥ 16%) benefited from rucaparib^91^. Similarly, 15% of sarcoma patients in the current cohort showed high gLOH (≥ 19.3%), warranting further evaluation of gLOH as a biomarker of response to DNA damage repair-targeted agents.

Our findings have many limitations. Unlike TCGA and other endeavors, pathology was not re-reviewed by a sarcoma pathologist, which may have led to some misclassifications. We also do not have information on stage, tumor grade, or whether the well or dedifferentiated component of WD/DD-LPS was submitted for sequencing. However, these real-world data provide a unique view of sarcoma pathology diagnosis outside tertiary cancer referral centers, where diagnostic errors are expected to be more frequent for rare cancers. Another limitation is the lack of matched normal control DNA, which could result in inadvertent inclusion of germline variants. While the gene set in this panel is extensive, it does not cover many genes that may be important in sarcomas such as *MYOD1, VEGFA, TERT, MTAP*, and *POLE/D* and many emerging pathways such as Hippo (*YAP1, TAZ*); underscoring the importance of gene panels to be continuously refined based on emerging knowledge. In addition, we recognize that some variants were found with low allele frequency and future studies are warranted to explore the impact of these variants on clonality and tumor heterogeneity. Lastly, as patients with refractory or advanced disease are more likely to have their tumors genomically profiled, this selection bias may have increased the observed frequency of alterations. As noted earlier, genomic profiling may be an aid to accurate and rapid diagnosis of sarcoma, but it should supplement, not replace, thoughtful pathologic review. The clinical utility of genomic profiling is currently being explored in a randomized, prospective trial in Europe (NCT03784014).

Taken together, these findings suggest a growing clinical utility for genomic sequencing, especially in the management of rare cancers such as sarcomas. In light of potential cost and resource limitations, a framework for judicious use of NGS testing should be developed for sarcomas, similar to the World Sarcoma Network recommendations for *NTRK* fusion testing^35^. The accompanying variant- and patient-level data provides a rich resource for future discoveries and clinical trial design in these rare cancers. Our data extend the growing body of evidence suggesting that genomics-based matching of patients to therapies has the potential to improve clinical outcomes for patients with sarcoma of soft tissue and bone.

## Methods

Following approval from institutional review boards (Western IRB 20152817 [full cohort], MSK 16–1101 [MSK cohort]), we retrospectively examined the genomic profiles of clinical sarcoma specimens analyzed by Foundation Medicine during the period 2012 through 2018. The requirement for informed consent was waived for both protocols. The Institutional Review Board granted a waiver of informed consent under 45 CFR § 46.116 based on review and determination that this research meets the following requirements: (i) the research involves no more than minimal risk to subjects; (ii) the research could not practicably be carried out without the requested waiver; (iii) the waiver will not adversely affect the rights and welfare of the subjects. All patients were assigned a diagnosis of sarcoma by the submitting clinician or by pathologist review of the test requisition form and the submitted pathology report, and profiling was initially performed in the course of routine clinical care using the FoundationOne® (Foundation Medicine, Inc., Cambridge, MA) or FoundationOne® Heme platform. Age, gender, anatomical location of tumor biopsy or resection, and histological subtype was collected, while stage, diagnostic pathology slides, and treatment outcomes were unavailable. For the MSK cohort, we identified 118 adult sarcoma patients (≥18 years of age) who underwent Foundation Medicine genomic profiling as part of their routine clinical care and collected clinical data from the electronic medical record. Patients were enrolled on clinical trials following written informed consent or treated with FDA-approved drugs (off-label) based on tumor genomics. No patient was treated with an investigational drug either as compassionate use or as single patient use (SPU). Details of clinical trials or off-label use of FDA approved drugs are provided in Supplementary Table S8. Radiographic images in Fig. 6 do not identify individuals and thus patient consent for publication was not required.

Following review of tumor purity of each formalin-fixed, paraffin-embedded tissue sample, ≥50 ng tumor DNA and ≥ 200 ng RNA per specimen were extracted in a Clinical Laboratory Improvement Amendment (CLIA)-certified laboratory. Using hybridization-capture, adaptor ligation-based libraries, this material was sequenced to high uniform coverage (>500X read coverage depth) for all coding exons of 465 genes and select introns of 31 genes; RNA from 333 genes was sequenced to detect rearrangements in cancer-related genes^28,92^. Reads from sequencing were mapped to the hg19 reference genome using the BWA aligner v0.5.9^93^ Subsequently, sequence metrics were collected and duplicate reads were removed using Picard 1.47 and Samtools 0.1.12a^94^. Local alignment was optimization was performed using GATK 1.0.4705^95^ and variant calling was limited to targeted genomic regions. Reads with mapping quality <25 and base calls with a quality ≤2 were discarded. Copy number amplifications and homozygous deletions were detected by obtaining a log-ratio profile of the sample by first normalizing the sequence coverage obtained at all exons and genome-wide SNPs against a process-matched normal control, correcting for GC bias, segmenting, then combining with allele frequencies at sequenced SNPs to estimate computational tumor purity and copy numbers of each segment using Gibbs sampling. Rearrangements were identified by analyzing chimeric read pairs. Rearrangements were called from RNA-seq by aligning to the refSeq human transcriptome refSeq, then re-aligning suboptimally mapped reads to the hg19 reference genome. Chimera clusters identified from DNA and RNA read pairs were filtered for repetitive sequences, and by distributing mapped positions to identify rearrangements, which were subsequently annotated according to the genomic loci of both clusters. Tissues with insufficient tumor or nucleic acid yield were excluded. In addition to known or likely pathogenic genomic alterations including short variants (base pair substitutions, and insertions/deletions), copy number alterations and fusions/rearrangements, microsatellite instability status (MSI) and tumor mutational burden (TMB) were also examined. Alterations were detected agnostic to germline or somatic origin. Biallelic alterations predictions included short variants with LOH of the wildtype allele, as determined by zygosity status; homozygous copy number deletions; or presence of ≥2 pathogenic alterations in a gene in a sample^96^. Mutational signatures were determined by analyzing all point mutations except known oncogenic driver alterations and predicted germline alterations and decomposing the count of alterations in a trinucleotide context into the 30 Catalogue of Somatic Mutations in Cancer (COSMIC) signatures ^36,49^. Signatures were aggregated to APOBEC (signatures 2 and 13), smoking (signature 4), BRCA (signature 3), mismatch repair (signatures 6,16,20, and 26), aging (signature 1), UV (signature 7), POLE (signature 10), and alkylating (signature 11). Mutational signatures were deemed to be dominant in a sample if the score for a mutational class was ≥0.4. TMB was determined by dividing the count of all base substitutions and indels in the coding region of targeted genes (including synonymous alterations) except known and likely pathogenic alterations, alterations predicted to be germline, alterations that were recurrently germline, known alterations in dbSNP, and alterations that show up recurrently in ExAC by the 1.2 Mb of sequenced DNA^56,97^. Microsatellite instability was measured from intronic homopolymer repeat loci with adequate coverage for length variability through principal component analysis^98^. Percent genome‐wide loss of heterozygosity (gLOH) was calculated from 22 autosomal chromosomes using the genome-wide copy number profile and minor allele fractions (AF) of the >3500 SNPs sequenced^46,91^. Using a comparative genomic hybridization-like method, we obtained a log-ratio profile of the sample by normalizing the sequence coverage against a process-matched normal control. This profile was segmented and interpreted using AFs of the sequenced SNPs to estimate copy number and minor allele count at each segment. LOH was called if the estimated copy number was not 0 but the minor allele count was 0 at a given segment. LOH segments that spanned ≥90% of a whole chromosome or chromosome arm and for regions in which LOH inference was ambiguous were excluded from calculation of percent gLOH.

Many sarcomas harbor pathognomonic translocations and genetic signatures^1^. When sequencing results were discordant with pathology records, these cases were flagged as potential diagnostic errors^1,2^ (Supplementary Table S3). “Potentially actionable mutations” were defined as those that have therapeutic implications, i.e. predict response or resistance to systemic therapy as per the OncoKB classification system (www.oncokb.org) of levels of actionability^57^ (June 8, 2021). Biomarkers for which there is only preclinical evidence were not considered actionable.

### Statistics

Data were presented using descriptive statistics for continuous variables and frequency counts for categorical variables. Differences were considered statistically significant when *p* < 0.05. For multiple hypothesis testing, the false discovery rate (FDR) method was used to adjust *p* values. Fisher’s exact test was used to compare univariate proportions and non-parametric Mann–Whitney *U* to compare continuous distributions. Statistical tests and computation were carried out using Python 2.7 (Python Software Foundation) or R 4.1.1 (R Foundation for Statistical Computing).

### Reporting summary

Further information on research design is available in the Nature Research Reporting Summary linked to this article.

## Supplementary information

Supplementary Information

Supplementary Dataset 1

Reporting Summary

## Data Availability

The sequencing data generated in this study is derived from clinical samples. The variant call data used in this study are provided in Supplementary Data 1; other data necessary to verify the reported results are provided as Source Data. The underlying raw sequencing data are not available for this manuscript or through any data agreements with Foundation Medicine. Patients were not consented for the release, sharing or distribution of the underlying sequencing data. Source data are provided with this paper.

## Source data

Source Data

## Code availability

Code to reproduce the figures is provided as a markdown R template at https://github.com/djinfmi/gounder_et_al_2022.
