# Supplementary material for: Clinical genomic profiling in the management of patients with soft tissue and bone sarcoma
Source: Nat Commun. 2022 Jun 15;13:3406. doi: 10.1038/s41467-022-30496-0 (PMC9200814; doi:10.1038/s41467-022-30496-0)
Supplement: Supplementary file 1 — Supplementary Information [file 41467_2022_30496_MOESM1_ESM.pdf]

Figure S1

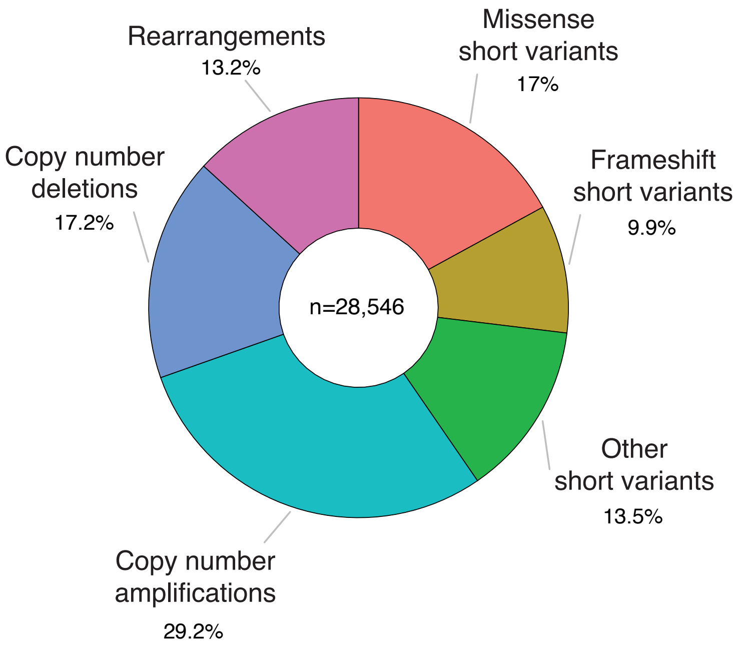

Supplementary Figure S1. Distribution of classes of genomic alterations across all sarcomas. A single tumor specimen may harbor multiple types of gene alterations. A total of 28,724 known or likely pathogenic variants (11,600 non-synonymous single nucleotide variants [SNVs]/indels, 13,287 copy number alterations, and 3,837 rearrangements) were detected.

Figure S2

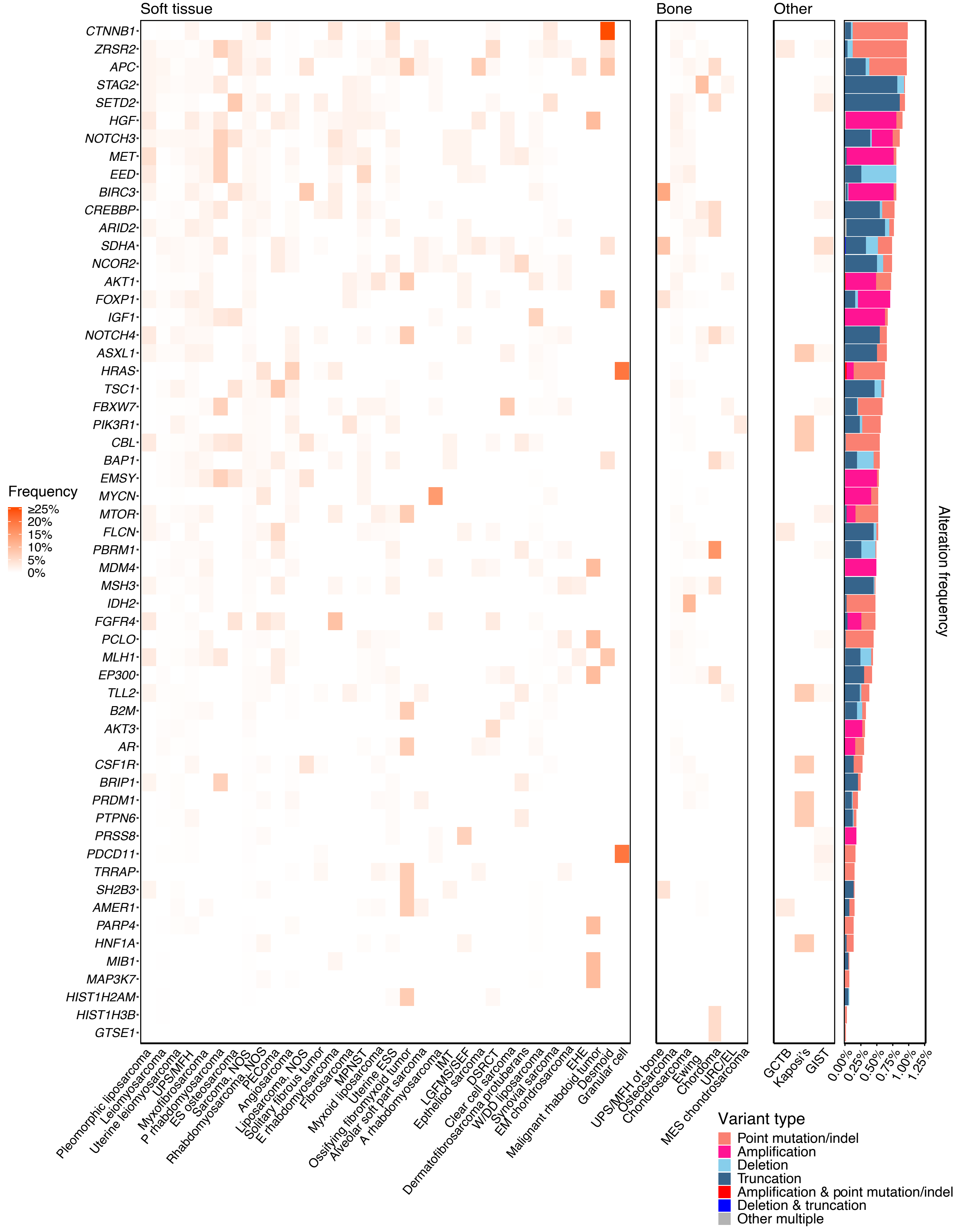

Soft tissue
Bone
Other
CTNNB1
ZRSR2
APC
STAG2
SETD2
HGF
NOTCH3
MET
EED
BIRC3
CREBBP
ARID2
SDHA
NCOR2
AKT1
FOXP1
IGF1
NOTCH4
ASXL1
HRAS
TSC1
FBXW7
PIK3R1
CBL
BAP1
EMSY
MYCN
MTOR
FLCN
PBRM1
MDM4
MSH3
IDH2
FGFR4
PCLO
MLH1
EP300
TLL2
B2M
AKT3
AR
CSF1R
BRIP1
PRDM1
PTPN6
PRSS8
PDCD11
TRRAP
SH2B3
AMER1
PARP4
HNF1A
MIB1
MAP3K7
HIST1H2AM
HIST1H3B
GTSE1
Frequency
≥25%
20%
15%
10%
5%
0%
Pleomorphic liposarcoma
Leiomyosarcoma
Uterine leiomyosarcoma
UPS/MFH
Myxofibrosarcoma
P rhabdomyosarcoma
ES osteosarcoma
Sarcoma, NOS
Rhabdomyosarcoma, NOS
PEComa
Angiosarcoma
Liposarcoma, NOS
Solitary fibrous tumor
E rhabdomyosarcoma
Fibrosarcoma
MPNST
Myxoid liposarcoma
Uterine ESS
Ossifying fibromyxoid tumor
Alveolar soft part sarcoma
A rhabdomyosarcoma
IMT
LGFMS/SEF
Epithelioid sarcoma
DSRCT
Clear cell sarcoma
Dermatofibrosarcoma protuberans
W/DD liposarcoma
Synovial sarcoma
EM chondrosarcoma
EHE
Malignant rhabdoid tumor
Desmoid
Granular cell
UPS/MFH of bone
Osteosarcoma
Chondrosarcoma
Ewing
Chordoma
URC/EL
MES chondrosarcoma
GCTB
Kaposi's
GIST
0.00%
0.25%
0.50%
0.75%
1.00%
1.25%
Alteration frequency
Variant type
Point mutation/indel
Amplification
Deletion
Truncation
Amplification & point mutation/indel
Deletion & truncation
Other multiple

Supplementary Figure S2. Heat map of recurrent somatic alterations across soft tissue, bone and other (GIST, Kaposi, giant cell tumor of bone, and rare) sarcomas. Genes with alteration frequency of < 1% in the total sarcoma cohort but ≥ 5% in any individual sarcoma subtype are listed along with the mechanisms of genetic alteration across tumor types. Right, percentage of cases with each type of genetic alteration.

Figure S3

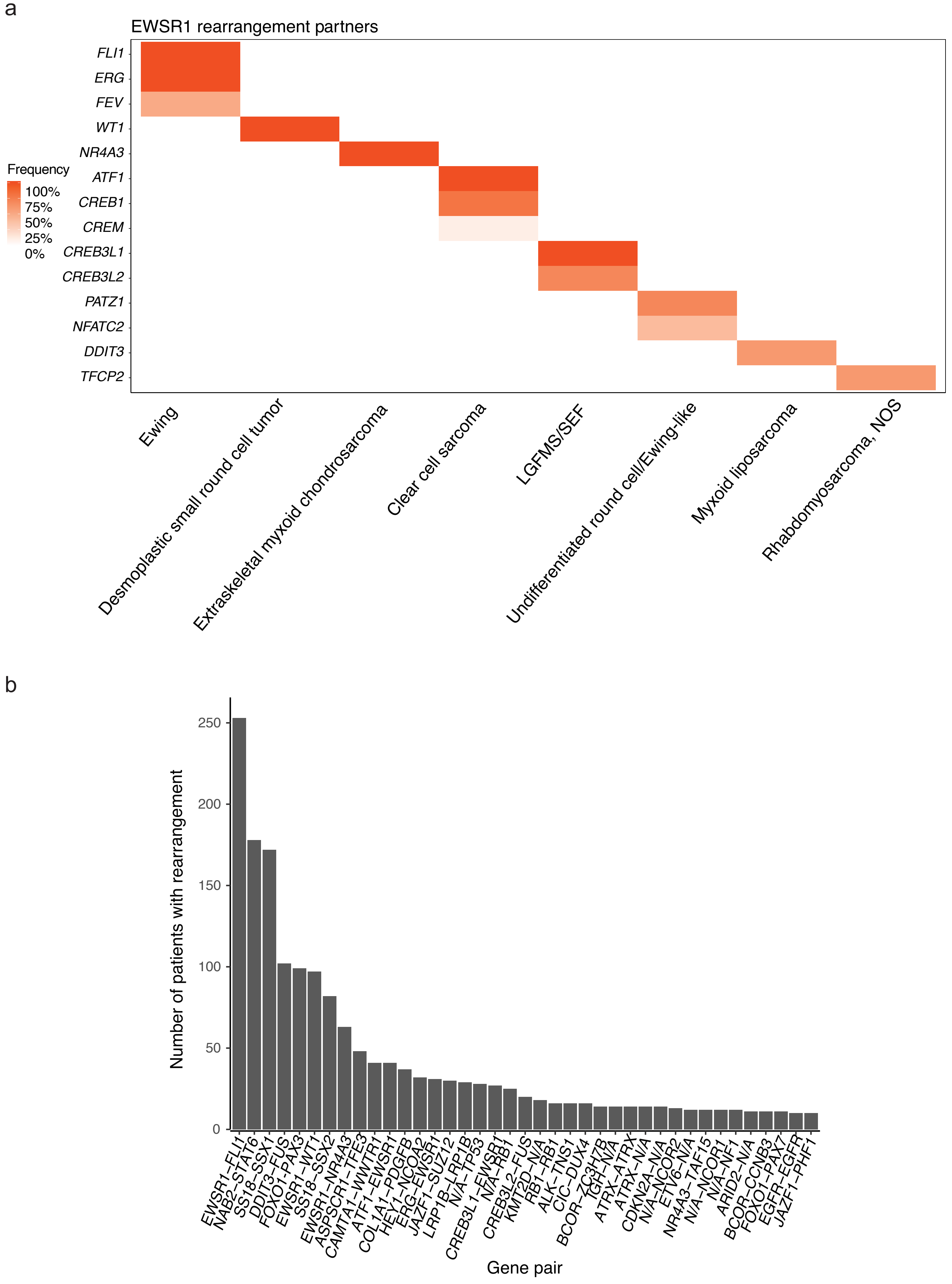

a
EWSR1 rearrangement partners
FLI1
ERG
FEV
WT1
NR4A3
ATF1
CREB1
CREM
CREB3L1
CREB3L2
PATZ1
NFATC2
DDIT3
TFCP2
Frequency
100%
75%
50%
25%
0%
Ewing
Desmoplastic small round cell tumor
Extraskeletal myxoid chondrosarcoma
Clear cell sarcoma
LGFMS/SEF
Undifferentiated round cell/Ewing-like
Myxoid liposarcoma
Rhabdomyosarcoma, NOS
b
Number of patients with rearrangement
250
200
150
100
50
0
EWSR1–FLI1
NAB2–STAT6
SS18–SSX1
DDIT3–FUS
FOXO1–PAX3
EWSR1–WT1
SS18–SSX2
EWSR1–NR4A3
ASPSCR1–TFE3
CAMTA1–WWTR1
ATF1–EWSR1
COL1A1–PDGFB
HEY1–NCOA2
ERG–EWSR1
JAZF1–SUZ12
LRP1B–LRP1B
N/A–TP53
CREB3L1–EWSR1
N/A–RB1
CREB3L2–FUS
KMT2D–N/A
RB1–RB1
ALK–TNS1
CIC–DUX4
BCOR–ZC3H7B
IGH–N/A
ATRX–ATRX
ATRX–N/A
CDKN2A–N/A
N/A–NCOR2
ETV6–N/A
NR4A3–TAF15
N/A–NCOR1
N/A–NF1
ARID2–N/A
BCOR–CCNB3
FOXO1–PAX7
EGFR–EGFR
JAZF1–PHF1
Gene pair

Supplementary Figure S3. Gene fusions. (a) *EWSR1* fusion partners **(b)** Distribution and frequency of all rearrangements across all sarcomas analyzed.

Figure S4

a

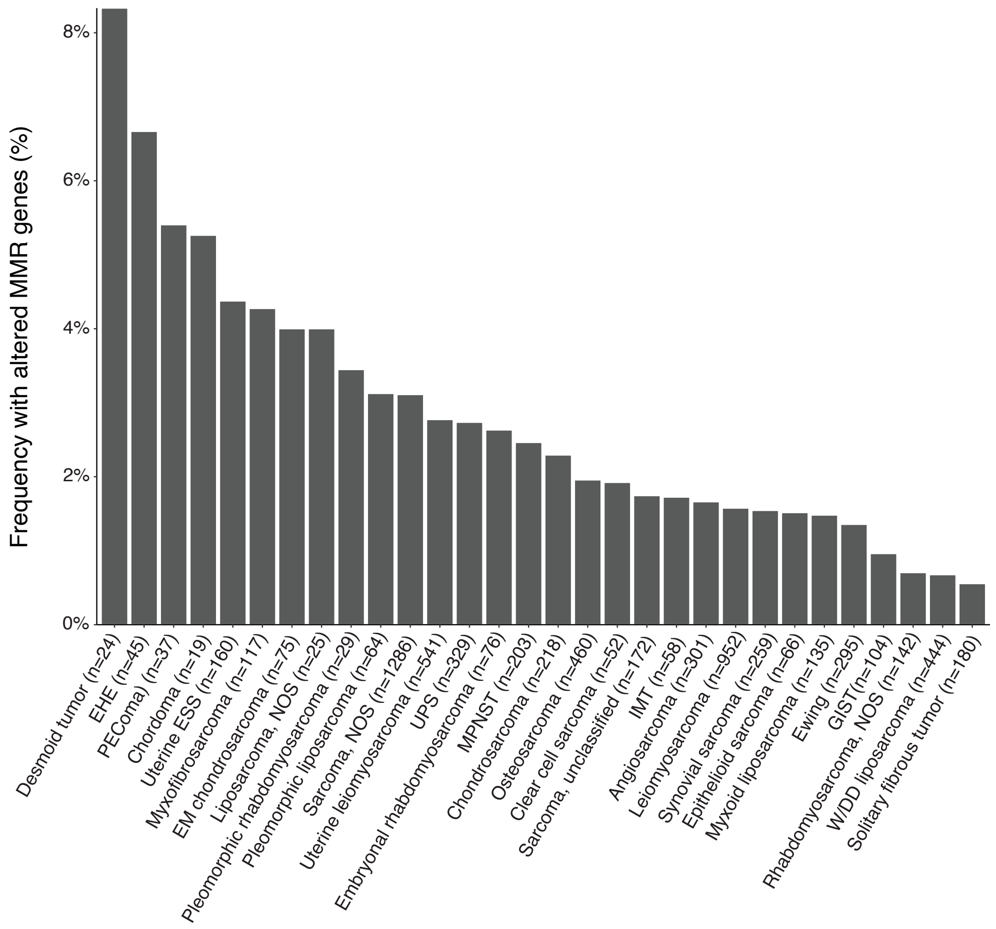
Frequency with altered MMR genes (%)
8%
6%
4%
2%
0%
Desmoid tumor (n=24)
EHE (n=45)
PEComa) (n=37)
Chordoma (n=19)
Uterine ESS (n=160)
Myxofibrosarcoma (n=117)
EM chondrosarcoma (n=75)
Liposarcoma, NOS (n=25)
Pleomorphic rhabdomyosarcoma (n=29)
Pleomorphic liposarcoma (n=64)
Sarcoma, NOS (n=1286)
Uterine leiomyosarcoma (n=541)
UPS (n=329)
Embryonal rhabdomyosarcoma (n=76)
MPNST (n=203)
Chondrosarcoma (n=218)
Osteosarcoma (n=460)
Clear cell sarcoma (n=52)
Sarcoma, unclassified (n=172)
IMT (n=58)
Angiosarcoma (n=301)
Leiomyosarcoma (n=952)
Synovial sarcoma (n=259)
Epithelioid sarcoma (n=66)
Myxoid liposarcoma (n=135)
Ewing (n=295)
GIST(n=104)
Rhabdomyosarcoma, NOS (n=142)
W/DD liposarcoma (n=444)
Solitary fibrous tumor (n=180)

b

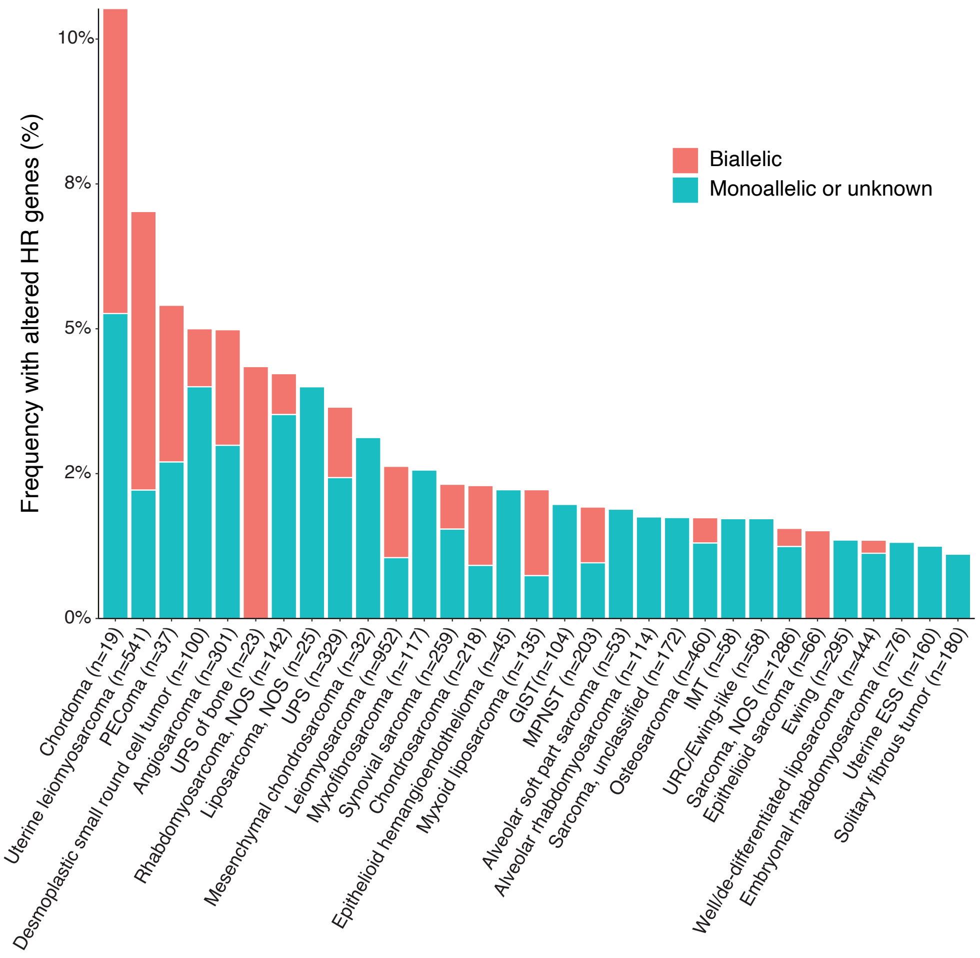
Frequency with altered HR genes (%)
10%
8%
5%
2%
0%
Biallelic
Monoallelic or unknown
Chordoma (n=19)
Uterine leiomyosarcoma (n=541)
PEComa (n=37)
Desmoplastic small round cell tumor (n=100)
Angiosarcoma (n=301)
UPS of bone (n=23)
Rhabdomyosarcoma, NOS (n=142)
Liposarcoma, NOS (n=25)
UPS (n=329)
Mesenchymal chondrosarcoma (n=32)
Leiomyosarcoma (n=952)
Myxofibrosarcoma (n=117)
Synovial sarcoma (n=259)
Chondrosarcoma (n=218)
Epithelioid hemangioendothelioma (n=45)
Myxoid liposarcoma (n=135)
GIST(n=104)
MPNST (n=203)
Alveolar soft part sarcoma (n=53)
Alveolar rhabdomyosarcoma (n=114)
Sarcoma, unclassified (n=172)
Osteosarcoma (n=460)
IMT (n=58)
URC/Ewing-like (n=58)
Sarcoma, NOS (n=1286)
Epithelioid sarcoma (n=66)
Ewing (n=295)
Well/de-differentiated liposarcoma (n=444)
Embryonal rhabdomyosarcoma (n=76)
Uterine ESS (n=160)
Solitary fibrous tumor (n=180)

Supplementary Figure S4. Alterations in mismatch repair and homologous recombination. **(a)** Distribution of the frequency of alterations in mismatch repair genes (irrespective of allelic status) among sarcomas. **(b)** Distribution of the frequency of alterations in homologous recombination genes among sarcomas.

Figure S5

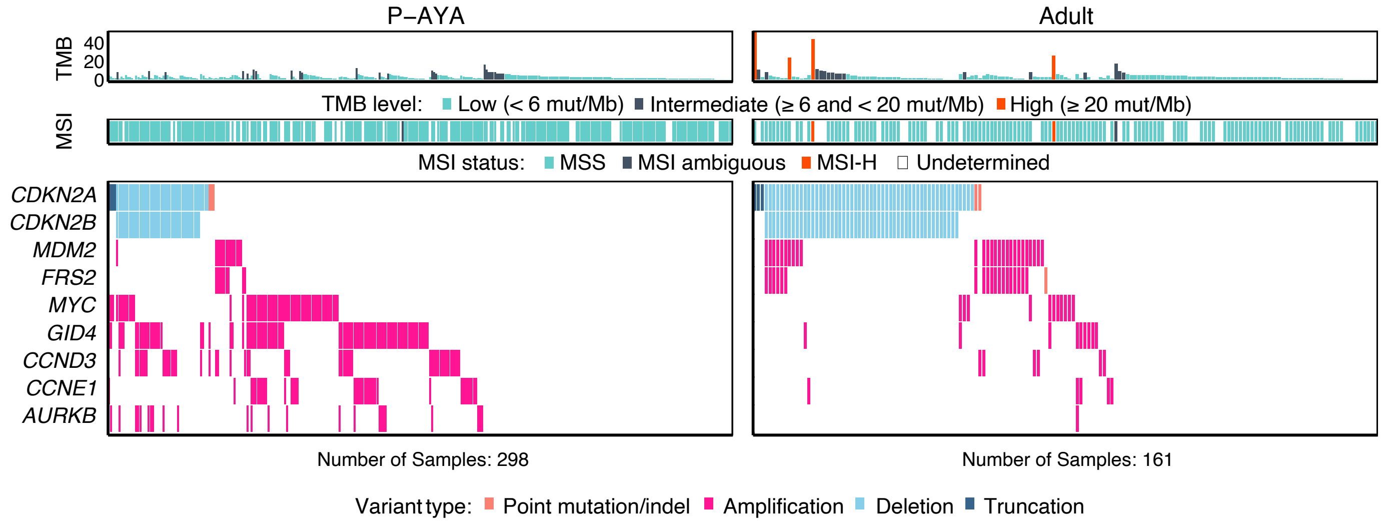

Supplementary Figure S5. Oncoplot of genomic alterations in pediatric, adolescent, and young adult patients versus adult patients diagnosed with osteosarcoma. Top row, tumor mutational burden (TMB) in mutations/Mb (mut/Mb). Pediatric and young adult (PAYA), ≤ 30 years of age; n = 298. Adults, n = 161.

Figure S6

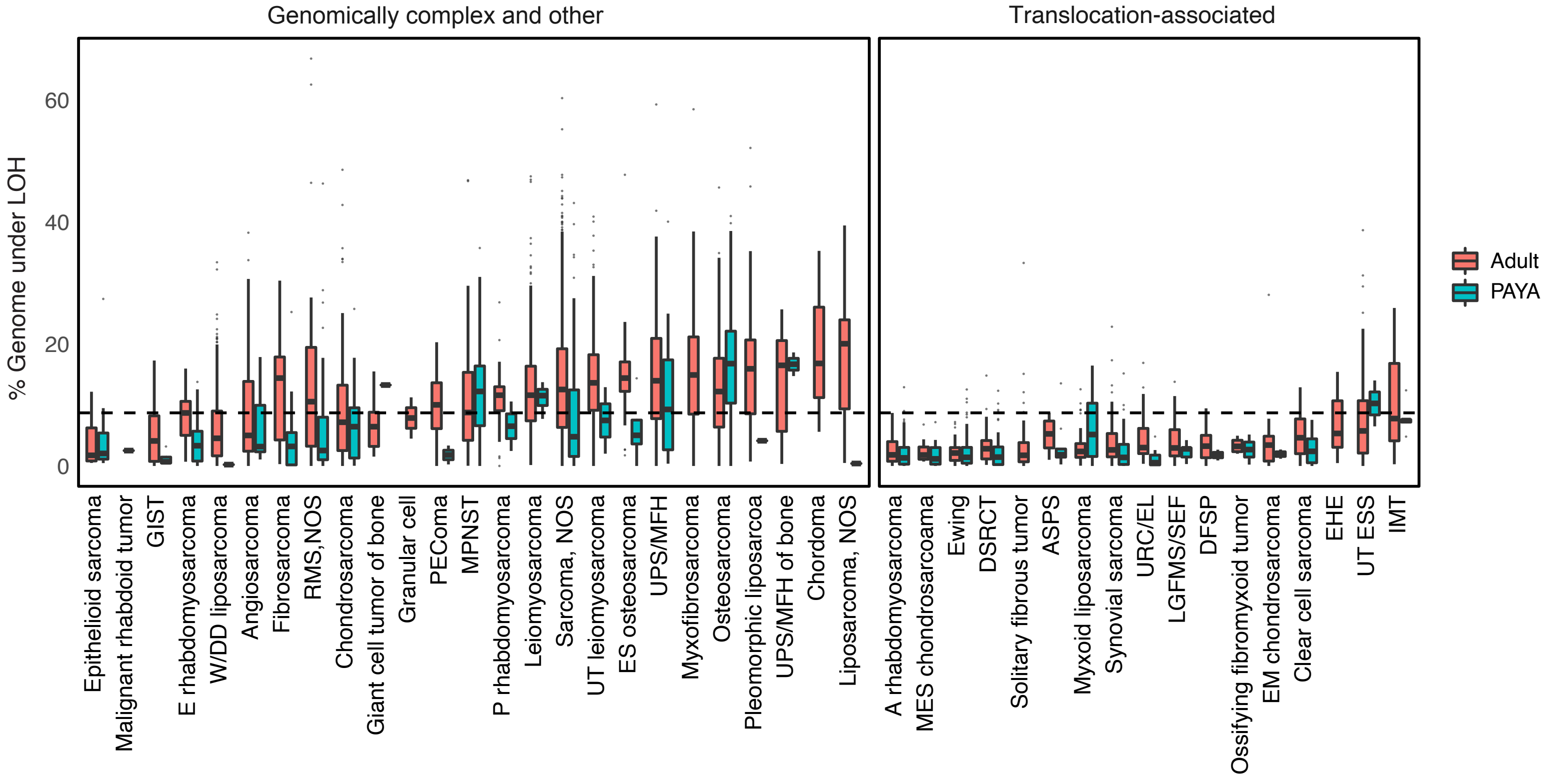

Supplementary Figure S6. Comparison of genomic loss of heterozygosity (gLOH) between P-AYA and adult patients within each sarcoma histology. gLOH only evaluable n = 4,619. The lower and upper boundary of the boxes represent the 25th and 75th percentiles; the line within the boxes represents the medians; whiskers extend to extreme values ≤ 1.5 x IQR; points beyond whiskers are outliers.

Supplementary Table S1. Targeted sequencing gene set. Exonic coverage (465 genes), intronic coverage (31 genes), andRNA sequencing coverage (333 genes).

**Exonic coverage: 465 genes**										
ABL1	BTG2	CSF3R	FANCC	GPR124	JAK3	MPL	PASK	RASGEF1A	SOCS3	U2AF2
ACTB	BTK	CTCF	FANCD2	GRAF	JARID2	MRE11A	PAX5	RB1	SOX10	VHL
AKT1	BTLA	CTNNA1	FANCE	GRIN2A	JUN	MSH2	PBRM1	REL	SOX2	WDR90
AKT2	C11orf30	CTNNB1	FANCF	GSK3B	KDM2B	MSH3	PC	RELN	SPEN	WHSC1
AKT3	C17orf39	CUL4A	FANCG	GTSE1	KDM4C	MSH6	PCBP1	RET	SPOP	WISP3
ALK	CAD	CUL4B	FANCI	HDAC1	KDM5A	MTOR	PCLO	RHOA	SRC	WT1
ALOX12B	CARD11	CUX1	FANCL	HDAC4	KDM5C	MUTYH	PDCD1	RICTOR	SRSF2	XBP1
APC	CASP8	CXCR4	FANCM	HDAC7	KDM6A	MYC	PDCD11	RMRP	STAG2	XPO1
APCDD1	CBFB	CYP17A1	FAT3	HGF	KDR	MYCL1	PDGFRA	RNF43	STAT3	XRCC3
APH1A	CBL	DAXX	FBXO11	HIST1H1C	KEAP1	MYCN	PDGFRB	ROS1	STAT4	YY1AP1
AR	CCND1	DDR2	FBXO31	HIST1H1D	KIT	MYD88	PDK1	RPA1	STAT5A	ZMYM3
ARAF	CCND2	DDX3X	FBXW7	HIST1H1E	KLHL6	MYO18A	PDL1	RPL11	STAT5B	ZNF217
ARFRP1	CCND3	DIS3	FGF10	HIST1H2AC	KRAS	MYST3	PDL2	RPL13	STAT6	ZNF703
ARID1A	CCNE1	DKC1	FGF12	HIST1H2AG	LEF1	NBN	PHF6	RPL15	STK11	ZRSR2
ARID2	CCT6B	DNM2	FGF14	HIST1H2AL	LMO1	NCOR1	PIK3C2G	RPL35A	SUFU	ZSCAN3
ASMTL	CD22	DNMT3A	FGF19	HIST1H2AM	LRP1B	NCOR2	PIK3C3	RPS14	SUZ12	
ASXL1	CD36	DOT1L	FGF23	HIST1H2BC	LRRK2	NCSTN	PIK3CA	RPS19	SYK	
ATM	CD58	DTX1	FGF3	HIST1H2BJ	MAF	NF1	PIK3CG	RPS26	TAF1	
ATR	CD70	DUSP2	FGF4	HIST1H2BK	MAFB	NF2	PIK3R1	RPTOR	TBL1XR1	
ATRX	CD79A	DUSP9	FGF6	HIST1H2BO	MAGED1	NFE2L2	PIK3R2	RUNX1	TBX3	
AURKA	CD79B	E2A	FGF7	HIST1H3B	MALT1	NFKBIA	PIM1	S1PR2	TCL1	
AURKB	CDC73	EBF1	FGFR1	HLA-A	MAP2K1	NKX2-1	PLCG2	SBDS	TET2	
AXIN1	CDH1	ECT2L	FGFR2	HNF1A	MAP2K2	NOD1	PMS2	SDHA	TGFBR2	
AXL	CDK12	EED	FGFR3	HRAS	MAP2K4	NOTCH1	PNRC1	SDHB	TIPARP	
B2M	CDK4	EGFR	FGFR4	HSP90AA1	MAP3K1	NOTCH2	POT1	SDHC	TLL2	
BACH1	CDK6	ELP2	FHIT	ICK	MAP3K13	NOTCH3	PPP2R1A	SDHD	TMEM30A	
BAP1	CDK8	EP300	FLCN	ID3	MAP3K14	NOTCH4	PRDM1	SERP2	TMSL3	

BARD1	CDKN1B	EPHA3	FLT1	IDH1	MAP3K6	NPM1	PRKAR1A	SETBP1	TNFAIP3	
BCL10	CDKN2A	EPHA5	FLT3	IDH2	MAP3K7	NRAS	PRKDC	SETD2	TNFRSF11A	
BCL11B	CDKN2B	EPHA7	FLT4	IGF1	MAPK1	NSD1	PRSS8	SF3B1	TNFRSF14	
BCL2	CDKN2C	EPHB1	FLYWCH1	IGF1R	MCL1	NT5C2	PTCH1	SGK1	TNFRSF17	
BCL2L2	CEBPA	ERBB2	FOXL2	IGF2	MDM2	NTRK1	PTEN	SH2B3	TNFRSF6	
BCL6	CHD2	ERBB3	FOXO1	IKBKE	MDM4	NTRK2	PTPN11	SHIP	TOP1	
BCL7A	CHEK1	ERBB4	FOXO3	IKZF1	MED12	NTRK3	PTPN2	SHP-1	TP53	
BCOR	CHEK2	ERG	FOXP1	IKZF2	MEF2B	NUP93	PTPRO	SMAD2	TP63	
BCORL1	CHUK	ESR1	FRS2	IKZF3	MEF2C	NUP98	RAD21	SMAD4	TRAF2	
BIRC3	CIC	ETO	GADD45B	IL7R	MEN1	P2RY8	RAD50	SMARCA1	TRAF3	
BLM	CIITA	ETS1	GATA1	INHBA	MET	PAG1	RAD51	SMARCA4	TRAF5	
BRAF	CKS1B	ETV6	GATA2	INPP4B	MIB1	PAK3	RAD51C	SMARCB1	TRRAP	
BRCA1	CPS1	EXOSC6	GATA3	IRF1	MITF	PAK7	RAD51L1	SMARCD1	TSC1	
BRCA2	CRBN	EZH2	GNA11	IRF4	MKI67	PALB2	RAD51L3	SMC1A	TSC2	
BRD4	CREBBP	FAF1	GNA12	IRF8	MLH1	PARP1	RAD52	SMC3	TSHR	
BRIP1	CRKL	FAM123B	GNA13	IRS2	MLL	PARP2	RAD54L	SMO	TUSC3	
BRSK1	CRLF2	FAM46C	GNAQ	JAK1	MLL2	PARP3	RAF1	SOCS1	TYK2	
BTG1	CSF1R	FANCA	GNAS	JAK2	MLL3	PARP4	RARA	SOCS2	U2AF1	

**Intronic coverage: 31 genes**										
ALK	BCR	CRLF2	ETV1	ETV6	IGH	JAK1	MYC	PDGFRB	RET	TRG
BCL2	BRAF	EGFR	ETV4	EWSR1	IGK	JAK2	NTRK1	RAF1	ROS1	
BCL6	CCND1	EPOR	ETV5	FGFR2	IGL	MLL	PDGFRA	RARA	TMPRSS2	

RNA sequencing coverage: 333 genes								
ABI1	BTG1	DEK	FOXO4	IRF4	MSN	PBX1	RET	TBL1XR1
ABL1	C1orf77	DLEU2	FOXP1	ITK	MTAP	PCM1	RHOH	TCL1
ABL2	CAMTA1	DNMT3A	FSTL3	JAK1	MTCP1	PCSK7	RNF213	TCL6
ACSL6	CARS	DUSP22	FUS	JAK2	MUC1	PDE4DIP	ROS1	TEC
AF10	CBFA2T3	E2A	GAS7	JAK3	MYB	PDGFB	RPA1	TET1
AF6	CBFB	EGFR	GLI1	JAZF1	MYC	PDGFRA	RPL13	TFE3
AFF1	CBL	EIF4A2	GLIS2	KDM4C	MYH11	PDGFRB	RPL15	TFG
AFF4	CCND1	ELF4	GMPS	KDSR	MYH9	PDK1	RPL22	TFPT
ALK	CCND2	ELL	GPHN	KIF5B	MYST3	PDL1	RPL35A	TFRC
ARHGEF12	CCND3	ELN	GRAF	LASP1	NACA	PDL2	RPN1	TLX1
ARID1A	CD247	EML4	HDAC4	LCK	NCOA2	PER1	RPS14	TLX3
ARID1B	CD70	ENL	HERPUD1	LCP1	NDRG1	PGAM5	RPS15	TMPRSS2
ARNT	CDC73	EP300	HEY1	LEF1	NF1	PHF1	RPS19	TNFRSF11A
ASXL1	CDK6	EPHA7	HIP1	LMO1	NF2	PICALM	RPS26	TNFSF9
ATF1	CDKN2A	EPOR	HIST1H1A	LMO2	NFKB2	PIK3R1	RUNDC2A	TOP1
ATG5	CDX2	EPS15	HIST1H4I	LPP	NFKBIE	PIK3R2	RUNX1	TP53
ATIC	CEBPA	ERBB2	HLF	LTK	NIN	PIM1	RUNX2	TP63
ATM	CEP110	ERG	HMGA1	LYL1	NKX2-1	PLAG1	SEC31A	TPM3
ATR	CHIC2	ETO	HMGA2	MAF	NOTCH1	PML	SEPT5	TPM4
ATXN1	CHN1	ETS1	HOXA11	MAFB	NPM1	POU2AF1	SEPT6	TRAF2
AXL	CIC	ETV1	HOXA13	MAGEA5	NR4A3	PPP1CB	SEPT9	TRAF3
BAP1	CIITA	ETV4	HOXA3	MALT1	NSD1	PRDM1	SET	TRAF5
BCL10	CKS1B	ETV5	HOXA9	MAP3K7	NTRK1	PRDM16	SH3GL1	TRG
BCL11A	CLP1	ETV6	HOXC11	MDS2	NTRK2	PRRX1	SLC1A2	TRIM24
BCL11B	CLTC	EWSR1	HOXC13	MECOM	NTRK3	PSIP1	SMARCB1	TRIP11
BCL2	CLTCL1	FBXW7	HOXD11	MEF2C	NUMA1	PTCH1	SRSF3	TSC1
BCL3	COL1A1	FCGR2B	HOXD13	MKL1	NUP214	PTEN	SS18	TSC2
BCL6	CREB3L1	FCRL4	HSP90AA1	MKL2	NUP98	PTK7	SSX1	TTL
BCL7A	CREB3L2	FEV	HSP90AB1	MLF1	NUTM2A	RABEP1	SSX2	TYK2

BCL8	CREBBP	FGFR1	IGH	MLL	OLIG2	RAF1	SSX4	USP6
BCL9	CRLF2	FGFR1OP	IGK	MLLT3	OMD	RALGDS	STAT6	WHSC1
BCOR	CSF1	FGFR2	IGL	MLLT6	P2RY8	RANBP17	STK11	WHSC1L1
BCR	CTNNB1	FGFR3	IKZF1	MN1	PAFAH1B2	RAP1GDS1	STL	YPEL5
BIRC3	DDIT3	FLI1	IKZF3	MNX1	PALB2	RARA	SYK	ZBTB16
BRAF	DDR1	FNBP1	IL21R	MSH2	PAX3	RB1	TAF15	ZMYM2
BRCA1	DDX10	FOXO1	IL3	MSH6	PAX5	RBM15	TAL1	ZNF384
BRCA2	DDX6	FOXO3	INSR	MSI2	PAX7	RCOR1	TAL2	ZNF521

Table S2. Categorization of sarcoma types.

Translocation-associated	Genomically complex or other
Ewing	Undifferentiated pleomorphic sarcoma/malignant fibrous histiocytoma
Solitary fibrous tumor	Uterine leiomyosarcoma
Myxoid liposarcoma	Leiomyosarcoma
Mesenchymal chondrosarcoma	Gastrointestinal stromal tumor
Uterine endometrial stromal sarcoma	Well-differentiated/dedifferentiated liposarcoma
Undifferentiated round cell/Ewing-like	Osteosarcoma
Alveolar soft part sarcoma	Embryonal rhabdomyosarcoma
Clear cell sarcoma	Sarcoma, NOS
Alveolar rhabdomyosarcoma	Chondrosarcoma
Synovial sarcoma	Epithelioid sarcoma
Inflammatory myofibroblastic tumor	Malignant peripheral nerve sheath tumor
Extraskeletal myxoid chondrosarcoma	Pleomorphic liposarcoma
Epithelioid hemangioendothelioma	Perivascular epithelioid cell tumor (PEComa)
Low-grade fibromyxoid sarcoma/ sclerosing epithelioid fibrosarcoma	Angiosarcoma
Desmoplastic small round cell tumor	Rhabdomyosarcoma, NOS
dermatofibrosarcoma protuberans	Kaposi’s sarcoma
Ossifying fibromyxoid tumor	Fibrosarcoma
	Myxofibrosarcoma
	Giant cell tumor of bone
	Extraskeletal osteosarcoma
	Pleomorphic rhabdomyosarcoma
	Chordoma
	Liposarcoma, NOS
	Undifferentiated pleomorphic sarcoma/malignant fibrous histiocytoma of bone
	Rare sarcoma
	Desmoid
	Malignant rhabdoid tumor
	Granular cell

Table S3. Subtype reclassifications based on genomic data. Any of the listed alterations was sufficient reason for reclassification. (P) presence; (A) absence (A).

From	To	Counts altered	Genomic basis
Osteosarcoma	Mesenchymal chondrosarcoma	5	(P): *HEY1-NCOA2*
Osteosarcoma	Synovial sarcoma	2	(P): *SSX18-SSX1/2/4*
Osteosarcoma	Low-grade fibromyxoid sarcoma/sclerosing epithelioid fibrosarcoma	2	(P): *EWSR1-CRB3L1/2*
Dermatofibrosarcoma protuberans	Sarcoma, unclassified	7	(A): *COL1A1-PDGFB*
Dermatofibrosarcoma protuberans	Solitary fibrous tumor	1	(P): *NAB2-STAT6*
Uterine endometrial stromal sarcoma	Rhabdomyosarcoma, NOS	1	(P): *FOXO1-FGFR1, PAX3-NCOA1, PAX3-NCOA2, EWSR1-TFCP2, MEIS1-NCOA2,* or *NAB2-PDGFRB*
Chondrosarcoma	Mesenchymal chondrosarcoma	24	(P): *HEY1-NCOA2*
Chondrosarcoma	Extraskeletal myxoid chondrosarcoma	68	(P): *NR4A3* with *EWSR1, FUS, TAF15, TCF12,* or *TFG*
Chondrosarcoma	Sarcoma, NOS	1	(P): *EWSR1-CREM or NAB2-PDGFRB*
Chondrosarcoma	Undifferentiated round cell sarcoma/Ewing-like	1	(P): *BCOR-CCNB3, CIC-DUX4, CIC-DUX4L10, CIC-FOXO4, EWSR1-SMARCA5, EWSR1-NFATC2, CIC-DUX4L-BCOR-MAML3, or EWSR1-PATZ1*
Chondrosarcoma	Myxoid liposarcoma	2	(P): *DDIT3-EWSR1 or DDIT3-FUS*
Chondrosarcoma	Solitary fibrous tumor	1	(P): *NAB2- STAT6*
Rare sarcoma	Extraskeletal myxoid chondrosarcoma	2	(P): *NR4A3 with EWSR1, FUS, TAF15, TCF12, or TFG*

Rare sarcoma	Undifferentiated round cell sarcoma/Ewing-like	2	(P): *BCOR-CCNB3, CIC-DUX4, CIC-DUX4L10, CIC-FOXO4, EWSR1-SMARCA5, EWSR1-NFATC2, CIC-DUX4L-BCOR-MAML3,* or *EWSR1-PATZ1*
Rare sarcoma	Ossifying fibromyxoid tumor	3	(P): *PHF1-EP400* or *PHF1-TFE3*
Rare sarcoma	Ewing	1	(P): *EWSR1* or *FUS* with *ERG, ETV1/4, FEV, FLI1* or *ERG*
Rare sarcoma	Sarcoma, unclassified	13	Withheld
Rare sarcoma	Rhabdomyosarcoma, NOS	1	(P): *FOXO1-FGFR1, PAX3-NCOA1, PAX3-NCOA2, EWSR1-TFCP2, MEIS1-NCOA2,* or *NAB2-PDGFRB*
Sarcoma, NOS	Extraskeletal myxoid chondrosarcoma	4	(P): *NR4A3* with *EWSR1, FUS, TAF15, TCF12,* or *TFG*
Sarcoma, NOS	Dermatofibrosarcoma protuberans	4	(P): *COL1A1-PDGFB*
Sarcoma, NOS	Inflammatory myofibroblastic tumor	3	(P): *EML4-ALK*
Sarcoma, NOS	Undifferentiated round cell sarcoma/Ewing-like	14	(P): *BCOR-CCNB3, CIC-DUX4, CIC-DUX4L10, CIC-FOXO4, EWSR1-SMARCA5, EWSR1-NFATC2, CIC-DUX4L-BCOR-MAML3,* or *EWSR1-PATZ1*
Sarcoma, NOS	Synovial sarcoma	24	(P): *SSX18-SSX1/2/4*
Sarcoma, NOS	Ossifying fibromyxoid tumor	2	(P): *PHF1-EP400* or *PHF1-TFE3*
Sarcoma, NOS	Ewing	6	(P): *EWSR1* or *FUS* with *ERG, ETV1/4, FEV, FLI1* or *ERG*
Sarcoma, NOS	Alveolar soft part sarcoma	4	(P): *ASPSCR1-TFE3*
Sarcoma, NOS	Low-grade fibromyxoid sarcoma/sclerosing epithelioid fibrosarcoma	21	(P): *EWSR1-CRB3L1/2*
Sarcoma, NOS	Alveolar rhabdomyosarcoma	2	(P): *PAX3* or *PAX7* with *FOXO1* or *FOXO4*
Sarcoma, NOS	Rhabdomyosarcoma, NOS	2	(P): *FOXO1-FGFR1, PAX3-NCOA1, PAX3-NCOA2, EWSR1-TFCP2, MEIS1-NCOA2, NAB2-PDGFRB*

Sarcoma, NOS	Myxoid liposarcoma	4	(P): *DDIT3-EWSR1* or *DDIT3FUS*
Sarcoma, NOS	Desmoplastic small round cell tumor	6	(P): *EWSR1-WT1*
Sarcoma, NOS	Solitary fibrous tumor	30	(P): *NAB2- STAT6*
Epithelioid hemangioendothelioma	Sarcoma, unclassified	25	(A): *WWTR1-CAMTA1* or *YAP1-TFE3*
Leiomyosarcoma	Mesenchymal chondrosarcoma	1	(P): *HEY1-NCOA2*
Leiomyosarcoma	Dermatofibrosarcoma protuberans	1	(P): *COL1A1-PDGFB*
Leiomyosarcoma	Inflammatory myofibroblastic tumor	1	(P): *ATIC-ALK, CARS-ALK, CLTC-ALK, EML4-ALK, FN1-ALK, LMNA-ALK, PPFIBP1-ALK, PRKAR1A-ALK, RANBP2-ALK, SEC31-ALK, TFG-ALK, TFG-ROS1, TPM3-ALK, TPM4-ALK, YWHAE-ROS1*
Leiomyosarcoma	Synovial sarcoma	2	(P): *SSX18-SSX1/2/4*
Leiomyosarcoma	Solitary fibrous tumor	1	(P): *NAB2-STAT6*
Liposarcoma, NOS	Well/de-differentiated liposarcoma	129	(P): *MDM2* amplification
Liposarcoma, NOS	Myxoid liposarcoma	8	(P): *DDIT3-EWSR1* or *DDIT3-FUS*
Epithelioid sarcoma	Low-grade fibromyxoid sarcoma/sclerosing epithelioid fibrosarcoma	1	(P): *EWSR1-CRB3L1/2*
Pleomorphic liposarcoma	Well/de-differentiated liposarcoma	11	(P): *MDM2* amplification
Angiosarcoma	Epithelioid hemangioendothelioma	1	(P): *WWTR1-CAMTA1* or *YAP1-TFE3*
Angiosarcoma	Undifferentiated round cell sarcoma/Ewing-like	1	(P): *BCOR-CCNB3, CIC-DUX4, CIC-DUX4L10, CIC-FOXO4, EWSR1-SMARCA5, EWSR1-NFATC2, CIC-DUX4L-BCOR-MAML3,* or *EWSR1-PATZ1*
Angiosarcoma	Synovial sarcoma	1	(P): *SSX18-SSX1/2/4*
Synovial sarcoma	Undifferentiated round cell sarcoma/Ewing-like	2	(A): *SSX18-SSX1/2/4*

Synovial sarcoma	Sarcoma, unclassified	44	(A): *SSX18-SSX1/2/4*
Synovial sarcoma	Low-grade fibromyxoid sarcoma/sclerosing epithelioid fibrosarcoma	2	(A): *SSX18-SSX1/2/4*
Synovial sarcoma	Rhabdomyosarcoma, NOS	1	(A): *SSX18-SSX1/2/4*
Synovial sarcoma	Solitary fibrous tumor	2	(P): *NAB2-STAT6*
Perivascular epithelioid cell tumor (pecoma)	Ewing	1	(P): *EWSR1* or *FUS* with *ERG, ETV1/4, FEV, FLI1* or *ERG*
Fibrosarcoma	Dermatofibrosarcoma protuberans	5	(P): *COL1A1-PDGFB*
Fibrosarcoma	Inflammatory myofibroblastic tumor	2	(P): *ATIC-ALK, CARS-ALK, CLTC-ALK, EML4-ALK, FN1-ALK, LMNA-ALK, PPFIBP1-ALK, PRKAR1A-ALK, RANBP2-ALK, SEC31-ALK, TFG-ALK, TFG-ROS1, TPM3-ALK, TPM4-ALK, YWHAE-ROS1*
Fibrosarcoma	Synovial sarcoma	1	(P): *SSX18-SSX1/2/4*
Fibrosarcoma	Low-grade fibromyxoid sarcoma/sclerosing epithelioid fibrosarcoma	21	*(P): EWSR1-CRB3L1/2*
Fibrosarcoma	Solitary fibrous tumor	1	(P): *NAB2-STAT6*
Ewing	Inflammatory myofibroblastic tumor	1	(P): *ATIC-ALK, CARS-ALK, CLTC-ALK, EML4-ALK, FN1-ALK, LMNA-ALK, PPFIBP1-ALK, PRKAR1A-ALK, RANBP2-ALK, SEC31-ALK, TFG-ALK, TFG-ROS1, TPM3-ALK, TPM4-ALK, YWHAE-ROS1*
Ewing	Undifferentiated round cell sarcoma/Ewing-like	14	(P): *BCOR-CCNB3, CIC-DUX4, CIC-DUX4L10, CIC-FOXO4, EWSR1-SMARCA5, EWSR1-NFATC2, CIC-DUX4L-BCOR-MAML3, or EWSR1-PATZ1*
Ewing	Synovial sarcoma	3	(P): *SSX18-SSX1/2/4*
Ewing	Sarcoma, unclassified	40	(A): *EWSR1* or *FUS* with *ERG, ETV1/4, FEV, FLI1* or *ERG*
Ewing	Desmoplastic small round cell tumor	8	(P): *EWSR1-WT1*

Well/de-differentiated liposarcoma	Pleomorphic liposarcoma	24	(P): *MDM2* amplification
Well/de-differentiated liposarcoma	Solitary fibrous tumor	1	(P): *NAB2-STAT6*
Undifferentiated pleomorphic sarcoma of bone	Undifferentiated round cell sarcoma/Ewing-like	1	(P): *BCOR-CCNB3, CIC-DUX4, CIC-DUX4L10, CIC-FOXO4, EWSR1-SMARCA5, EWSR1-NFATC2, CIC-DUX4L-BCOR-MAML3,* or *EWSR1-PATZ1*
Undifferentiated pleomorphic sarcoma of bone	Ewing	1	(P): *EWSR1* or *FUS* with *ERG, ETV1/4, FEV, FLI1* or *ERG*
Undifferentiated pleomorphic sarcoma of bone	Solitary fibrous tumor	1	(P): *NAB2-STAT6*
Malignant peripheral nerve sheath tumor (mpnst)	Synovial sarcoma	3	(P): *SSX18-SSX1/2/4*
Malignant peripheral nerve sheath tumor (mpnst)	Low-grade fibromyxoid sarcoma/sclerosing epithelioid fibrosarcoma	1	(P): *EWSR1-CRB3L1/2*
Alveolar soft part sarcoma	Sarcoma, unclassified	11	(A): *ASPSCR1-TFE3*
Alveolar soft part sarcoma	Low-grade fibromyxoid sarcoma/sclerosing epithelioid fibrosarcoma	1	(P): *EWSR1-CRB3L1/2*<br>(A): *ASPSCR1-TFE3*
Uterine leiomyosarcoma	Dermatofibrosarcoma protuberans	2	(P): *COL1A1-PDGFB*
Uterine leiomyosarcoma	Uterine endometrial stromal sarcoma	3	(P): *BCOR:ZC3H7B* (and uterus primary)
Uterine leiomyosarcoma	Inflammatory myofibroblastic tumor	1	(P): *ATIC-ALK, CARS-ALK, CLTC-ALK, EML4-ALK, FN1-ALK, LMNA-ALK, PPFIBP1-ALK, PRKAR1A-ALK, RANBP2-ALK, SEC31-ALK, TFG-ALK, TFG-ROS1, TPM3-ALK, TPM4-ALK, YWHAE-ROS1*
Uterine leiomyosarcoma	Solitary fibrous tumor	1	(P): *NAB2-STAT6*
Undifferentiated pleomorphic sarcoma	Mesenchymal chondrosarcoma	1	(P): *HEY1-NCOA2*

Undifferentiated pleomorphic sarcoma	Inflammatory myofibroblastic tumor	2	(P): *ATIC-ALK, CARS-ALK, CLTC-ALK, EML4-ALK, FN1-ALK, LMNA-ALK, PPFIBP1-ALK, PRKAR1A-ALK, RANBP2-ALK, SEC31-ALK, TFG-ALK, TFG-ROS1, TPM3-ALK, TPM4-ALK, YWHAE-ROS1*
Undifferentiated pleomorphic sarcoma	Undifferentiated round cell sarcoma/Ewing-like	2	(P): *BCOR-CCNB3, CIC-DUX4, CIC-DUX4L10, CIC-FOXO4, EWSR1-SMARCA5, EWSR1-NFATC2, CIC-DUX4L-BCOR-MAML3,* or *EWSR1-PATZ1*
Undifferentiated pleomorphic sarcoma	Ewing	2	(P): *EWSR1* or *FUS* with *ERG, ETV1/4, FEV, FLI1* or *ERG*
Undifferentiated pleomorphic sarcoma	Low-grade fibromyxoid sarcoma/sclerosing epithelioid fibrosarcoma	1	(P): *EWSR1-CRB3L1/2*
Undifferentiated pleomorphic sarcoma	Rhabdomyosarcoma, NOS	1	(P): *FOXO1-FGFR1, PAX3-NCOA1, PAX3-NCOA2, EWSR1-TFCP2, MEIS1-NCOA2, NAB2-PDGFRB*
Undifferentiated pleomorphic sarcoma	Desmoplastic small round cell tumor	1	(P): *EWSR1-WT1*
Undifferentiated pleomorphic sarcoma	Solitary fibrous tumor	2	(P): *NAB2-STAT6*
Myxofibrosarcoma	Low-grade fibromyxoid sarcoma/sclerosing epithelioid fibrosarcoma	4	(P): *EWSR1-CRB3L1/2*
Embryonal rhabdomyosarcoma	Alveolar rhabdomyosarcoma	5	(P): *PAX3* or *PAX7* with *FOXO1* or *FOXO4*
Embryonal rhabdomyosarcoma	Rhabdomyosarcoma, NOS	2	(P): *FOXO1-FGFR1, PAX3-NCOA1, PAX3-NCOA2, EWSR1-TFCP2, MEIS1-NCOA2,* or *NAB2-PDGFRB*
Clear cell sarcoma	Ewing	1	(P): *EWSR1* or *FUS* with *ERG, ETV1/4, FEV, FLI1* or *ERG*
Clear cell sarcoma	Sarcoma, unclassified	5	(A): *EWSR1* and *CREB1* or *ATF1*
Alveolar rhabdomyosarcoma	Undifferentiated round cell sarcoma/Ewing-like	1	(P): *BCOR-CCNB3, CIC-DUX4, CIC-DUX4L10, CIC-FOXO4, EWSR1-SMARCA5, EWSR1-NFATC2, CIC-DUX4L-BCOR-MAML3,* or *EWSR1-PATZ1*

Alveolar rhabdomyosarcoma	Alveolar soft part sarcoma	1	(P): *ASPSCR1-TFE3*
Alveolar rhabdomyosarcoma	Rhabdomyosarcoma, NOS	16	(P): *PAX3* or *PAX7* with *FOXO1* or *FOXO4*
Alveolar rhabdomyosarcoma	Desmoplastic small round cell tumor	1	(P): *EWSR1-WT1*
Rhabdomyosarcoma, NOS	Mesenchymal chondrosarcoma	1	(P): *HEY1-NCOA2*
Rhabdomyosarcoma, NOS	Alveolar rhabdomyosarcoma	36	(P): *PAX3* or *PAX7* with *FOXO1* or *FOXO4*
Myxoid liposarcoma	Extraskeletal myxoid chondrosarcoma	1	(P): *DDIT3-EWSR1* or *DDIT3-FUS*
Myxoid liposarcoma	Well/de-differentiated liposarcoma	21	(P): *DDIT3-EWSR1* or *DDIT3-FUS*
Desmoplastic small round cell tumor	Undifferentiated round cell sarcoma/Ewing-like	2	(P): *EWSR1-WT1*
Desmoplastic small round cell tumor	Ossifying fibromyxoid tumor	1	(P): *EWSR1-WT1*
Desmoplastic small round cell tumor	Ewing	1	(P): *EWSR1-WT1*
Desmoplastic small round cell tumor	Sarcoma, unclassified	7	(P): *EWSR1-WT1*
Solitary fibrous tumor	Synovial sarcoma	2	(P): *SSX18-SSX1/2/4*
Solitary fibrous tumor	Sarcoma, unclassified	20	(P): *NAB2-STAT6*
Solitary fibrous tumor	Low-grade fibromyxoid sarcoma/sclerosing epithelioid fibrosarcoma	4	(P): *EWSR1-CRB3L1/2*

Table S4. Odd ratios of coincident alterations of two genes in the p53 or Rb pathways. P values determined by two-tailed Fisher's exact test; false discovery rate accounts for multiple hypothesis correction.

Gene1	Gene2	OR	P value	FDR
TP53	MDM2	0.04767393	6.93E-130	3.46E-129
TP53	MDM4	0.2717202	0.00293975	0.00419964
TP53	CDKN2A	0.83220995	0.00480173	0.00600216
TP53	CDKN2B	0.68454764	2.96E-07	9.87E-07
MDM2	MDM4	3.69810845	0.00097421	0.00162368
MDM2	CDKN2A	0.57544849	8.66E-07	2.16E-06
MDM2	CDKN2B	0.60210064	5.49E-05	0.00010973
CDKN2A	CDKN2B	3975.82676	0	0
RB1	CDKN2A	0.07003602	4.34E-80	7.81E-79
RB1	CDKN2B	0.07001102	9.87E-61	1.18E-59
RB1	CDK4	0.04295226	2.86E-56	2.57E-55
RB1	CCND1	0.2042624	0.00148076	0.00332102
RB1	CCND2	0.23994812	0.0002073	0.00062189
RB1	CDK6	0.09541085	0.0012272	0.00315565
CDKN2A	CDKN2B	3975.82676	0	0
CDKN2A	CDK4	0.3416311	1.32E-17	7.90E-17
CDKN2A	CCNE1	0.32041248	1.46E-05	5.27E-05
CDKN2A	CCND1	1.89567508	0.01798866	0.03083771
CDKN2A	CCND2	1.76469653	0.01633936	0.02941085
CDKN2A	CCND3	2.13548723	0.00016025	0.00052445
CDKN2B	CDK4	0.36145797	5.97E-13	3.07E-12
CDKN2B	CCNE1	0.40099169	0.00148176	0.00332102
CDKN2B	CCND2	2.19009049	0.00156826	0.00332102
CDKN2B	CCND3	2.15831743	0.00038619	0.00106945
CDK4	CCND1	6.07022344	1.12E-11	5.04E-11
CDK4	CCND2	8.9461642	3.44E-24	2.47E-23
CDK4	CCND3	3.96195862	4.55E-11	1.82E-10
CCND2	CCND3	3.44613194	0.01098392	0.02081164
CCND3	CDK6	6.25128205	0.00194769	0.00389538

Table S5. Demographics and sequencing characteristics of MSK cohort (n = 118).

	n, %	Median, range
Age at diagnosis (years)		50 (12–89)
Gender		
Male	59 (50%)	
Female	59 (50%)	
Stage at diagnosis (n = 118)		
Stage I	6 (5.1%)	
Stage II	64 (54%)	
Stage III	12 (10%)	
Stage IV	36 (31%)	
Number of systemic therapies administered for metastatic or locally recurrent disease		4 (0-12)
Number of surgeries		2 (0-9)
Number of clinical trials enrolled per patient who went on systemic therapy		1 (0-5)
Histological subtypes		
Soft tissue	93 (79%)	
Leiomyosarcoma	16 (14%)	
Liposarcoma	15 (13%)	
Dedifferentiated	12 (10%)	
Myxoid	2 (1.7%)	
Pleiomorphic	1 (0.8%)	
Undifferentiated pleiomorphic sarcoma	13 (11%)	
Solitary fibrous tumor	6 (5.1%)	
PEComa	5 (4.2%)	
Synovial sarcoma	5 (4.2%)	
GIST	4 (3.4%)	
Angiosarcoma	4 (3.4%)	
MPNST	3 (2.5%)	
Intimal sarcoma	3 (2.5%)	
Other	19 (16%)	
Bone	25 (21%)	
Chordoma	9 (7.6%)	
Chondrosarcoma	9 (7.6%)	
Osteosarcoma	6 (5.1%)	
Ewing sarcoma	1 (0.8%)	

**Sequencing Characteristics**	
Time to Tumor sequencing from **initial diagnosis**, median (n = 106)	2.45 years (Range 0 – 23.5 years)

Time between **need/initiation of systemic therapy** and **consenting for genomic profiling**, median (n = 91)	1.1 years<br><br>(Range 0 – 11.8 years)
Number of FM Reports per year (n = 118)***:	
2012	9 (7.6%)
2013	54 (45.8%)
2014	40 (33.9%)
2015	14 (11.9%)
2016	1 (0.8%)
Sequencing Platform:	
v1	13 (11%)
v2	37 (31.4%)
v3	19 (16.1%)
v4	48 (40.7%)
v5	1 (0.8%)
Success rate of tumor sequencing:	
Successful	106 (91.0%)
Insufficient DNA or no alterations identified	12 (9.0%)
Tumor tissue sequenced (n = 106):	
Primary	43 (40.6%)
Recurrence	22 (20.7%)
Metastatic	41 (38.7%)
Chronology of tumor sequencing (ratio*):	
Early (0 – 0.33)	30 (33.0%)
Intermediate (0.33 – 0.66)	30 (33.0%)
Late (>0.66)	31 (34.0%)

Enrolled in <u>any</u> clinical trial **	
Early	18/30 (60.0%)
Intermediate	17/30 (57.0%)
Late	18/31 (58.0%)

* Ratio between time from start of systemic therapy to sequencing/Time from start of systemic therapy to death or last alive.

** Matched or unmatched clinical trials.

***For failed/insufficient reports, date of notice of failure was used.

Three patients (Figure 6e, 6f and 6g) are not part of the initial 118 MSKCC patients and included to illustrate as anecdotal examples.

Supplementary Table S6. Tumor characteristics and treatment of patients of 31 MSK patients matched to treatment based on targeted sequencing results.

Unique patients	Pathology	Biomarker	Clinical trial or agent	Trial number	PMID/PMC
1	Sarcoma, NOS	SMARCB1 del	A Phase II, Multicenter Study of the EZH2 Inhibitor Tazemetostat in Adult Subjects With INI1-Negative Tumors or Relapsed/Refractory Synovial Sarcoma and advanced cancer	NCT02601950	33035459
2	Myxoid Liposarcoma	PIK3CA G364R	An Open-Label, Phase Ib Dose Escalation Trial of Oral Combination Therapy with MSC1936369B and SAR245409 in Subjects with Locally Advanced or Metastatic Solid Tumors	NCT01390818	30425349
3	Sarcoma, NOS	PIK3CA E545A	A Phase 1, Open-Label, Multiple-Escalating-Dose Study of DS-7423, an Orally Administered Dual PI3K/mTOR Inhibitor, in Subjects with Advanced Solid Tumors	NCT01364844	29795308
4	Intimal Sarcoma	CDK4 amplification and RB1 wild-type	A Phase 1 Multicenter, Open Label, Dose-escalation Study of Oral LEE011 in Patients with Advanced Solid Tumors or Lymphomas	NCT01390818	5621377
4	Intimal Sarcoma	MDM2 amplification and TP53 wild-type	A Phase 1 Study to Assess the Safety, Tolerability, Pharmacokinetics, and Biological Activity of SAR405838 in Patients with Advanced Cancer	NCT01390818	27576846
5	UPS/MFH	CRKL amplification	Phase I Study of Dasatinib in Combination with Ipilimumab for Patients with Advanced Gastrointestinal Stromal Tumor and Other Sarcomas (NCI #9172)	NCT01643278	28007774
6	Liposarcoma	CDK4 amplification and RB1 wild-type	A Phase II Study of PD0332991 in Patients with Advanced or Metastatic Sarcoma	NCT01209598	23569312
6	Liposarcoma	MDM2 amplification and TP53 wild-type	A Phase 1 Multiple Ascending Dose Study of DS3032b in Subjects With Advanced Solid Tumors or Lymphomas	NCT01877382	ASCO 2018
7	GIST	KIT M552_W557del	Imatinib	NA	NA
8	Chordoma	MET amp	Crizotinib (Off-label use of FDA-approved)	NA	NA
9	Osteosarcoma	CRKL amp	Dasatinib (Off -abel use of FDA-approved)	NA	NA

10	Liposarcoma	CDK4 amplification RB1 wild-type	A Phase II Study of PD0332991 in Patients with Advanced or Metastatic Sarcoma	NCT01209598	23569312
10	Liposarcoma	MDM2 amplification and TP53 wild-type	A Phase 1 Study to Assess the Safety, Tolerability, Pharmacokinetics, and Biological Activity of SAR405838 in Patients with Advanced Cancer	NCT01390818	27576846
11	Liposarcoma	CDK4 amplification and RB1 wild-type	A Phase II Study of PD0332991 in Patients with Advanced or Metastatic Liposarcoma	NCT01209598	23569312
11	Liposarcoma	MDM2 amplification and TP53 wild-type	A Phase 1 Study to Assess the Safety, Tolerability, Pharmacokinetics, and Biological Activity of SAR405838 in Patients with Advanced Cancer	NCT01390818	27576846
12	Leiomyosarcoma/PEComa	TSC2 alteration	A Phase 1 First-in-Human Dose Study of LY3023414 an Oral PI3K/mTOR Dual Inhibitor in advanced tumors	NCT01655225	29636360
13	Chordoma	MDM2 amplification and TP53 wild-type	A Phase 1 Multiple Ascending Dose Study of DS3032b in Subjects With Advanced Solid Tumors or Lymphomas	NCT01877382	ASCO 2018
14	Liposarcoma	CDK4 amplification and RB1 wild-type	A Phase II Study of PD0332991 in Patients with Advanced or Metastatic Liposarcoma	NCT01209598	23569312
15	Leiomyosarcoma	CDK4 amplification RB1 wild-type	Palbociclib (off-label, FDA-approved drug)	NA	NA

16	Chondrosarcoma	IDH1 R132C	A Phase 1, Multicenter, Open-Label, Dose Escalation and Expansion, Safety, Pharmacokinetic, Pharmacodynamic, and Clinical Activity Study of Orally Administered AG-120 in Subjects with Advanced Solid Tumors, Including Glioma, with an IDH1 Mutation	NCT02073994	32208957
17	Myoepethelial carcinoma of soft tissue	SMARCB1 del	A Phase II, Multicenter Study of the EZH2 Inhibitor Tazemetostat in Adult Subjects With INI1-Negative Tumors or Relapsed/Refractory Synovial Sarcoma and advanced cancer	NCT02601950	33035459
18	MPNST	BRAF V600E	Dabrafenib and Trametinib (Off label use of FDA approved drug, treated at outside hospital with no detailed records)	NA	NA

19	Chondrosarcoma	IDH1 R132C	A Phase 1, Multicenter, Open-Label, Dose Escalation and Expansion, Safety, Pharmacokinetic, Pharmacodynamic, and Clinical Activity Study of Orally Administered AG-120 in Subjects with Advanced Solid Tumors, Including Glioma, with an IDH1 Mutation	NCT02073994	32208957
20	GIST	KIT D820V	Sunitinib (first line, off-label use of FDA approved drug)	NA	NA
21	MPNST	NTRK3-TPM4 fusion	A Phase 2 Basket Study of the Oral TRK Inhibitor larotrectinib in Subjects with NTRK Fusion-Positive Tumors	NCT02576431	29466156
22	Liposarcoma	CDK4 amplification and RB1 wild-type	A Phase II Study of PD0332991 in Patients with Advanced or Metastatic Liposarcoma	NCT01209598	23569312
22	Liposarcoma	CDK4 amplification and RB1 wild-type	A Phase II Study of PD0332991 in Patients with Advanced or Metastatic Liposarcoma	NCT01209598	23569312
23	PEComa	TSC2 Q1605	Sirolimus (off-label, FDA approved)	NA	NA

24	Follicular Dendritic Cell Sarcoma	FGFR3	A Randomized Discontinuation Study of Brivanib Alaninate (BMS-582664) Versus Placebo in Subjects with Advanced Tumors	NCT00633789	31522033
24	Follicular Dendritic Cell Sarcoma	FGFR3	A Phase I, Gene Alteration-Based, Open Label, Multicenter Study of Oral Debio1347 (CH5183284) in Patients with Advanced Solid Malignancies, Whose Tumors Have an Alteration of the FGFR 1, 2 or 3 Genes	NCT01948297	Unpublished
25	Chondrosarcoma	CDKN2A/B deletion	A Phase 1 Multicenter, Open Label, Dose-escalation Study of Oral LEE011 in Patients with Advanced Solid Tumors or Lymphomas	NCT01237236	27542767
26	Intimal Sarcoma	MDM2 amplification and TP53 wild-type	A Phase 1 Multiple Ascending Dose Study of DS3032b in Subjects With Advanced Solid Tumors or Lymphomas	NCT01877382	ASCO 2018
26	Intimal Sarcoma	CDK4 amplification and RB1 wild-type	Palbociclib (off label, FDA approved)	NA	NA
27	Liposarcoma	MDM2	A Phase 1 Multiple Ascending Dose Study of DS3032b in Subjects With Advanced Solid Tumors or Lymphomas	NCT01877382	ASCO 2018
28	Chordoma	CDKN2A/B del	Palbociclib (off label, FDA approved)	NA	NA
29	Leiomyosarcoma	TSC2 truncation	Everolimus (off label, FDA approved)	NA	NA

30	Liposarcoma	CDK4 amplification and RB1 wild-type	A Phase II Study of PD0332991 in Patients with Advanced or Metastatic Liposarcoma	NCT01209598	23569312
30	Liposarcoma	MDM2 amplification and TP53 wild-type	A Phase 1 Study to Assess the Safety, Tolerability, Pharmacokinetics, and Biological Activity of SAR405838 in Patients with Advanced Cancer	NCT01390818	27576846
31	Liposarcoma	MDM2 amplification and TP53 wild-type	A Phase I, Open Label, Multicenter, Dose-escalation Study of Oral HDM201 in Adult Patients with Advanced Solid and Hematological Tumors Characterized by Wild-type TP53	NCT02143635	Unpublished

Note: patients with more than 1 biomarker enrolled to different trials were only counted once.
