# Supplementary material for: Clinical genomic profiling in the management of patients with soft tissue and bone sarcoma
Source: Nat Commun. 2022 Jun 15;13:3406. doi: 10.1038/s41467-022-30496-0 (PMC9200814; doi:10.1038/s41467-022-30496-0)
Supplement: Supplementary file 3 — Reporting Summary [file 41467_2022_30496_MOESM3_ESM.pdf]

nature research

Corresponding author(s): Mrinal M. Gounder

Last updated by author(s): Mar 9, 2022

Reporting Summary

Nature Research wishes to improve the reproducibility of the work that we publish. This form provides structure for consistency and transparency in reporting. For further information on Nature Research policies, see Authors & Referees and the Editorial Policy Checklist.

Statistics

For all statistical analyses, confirm that the following items are present in the figure legend, table legend, main text, or Methods section.

n/a	Confirmed	
☐	☒	The exact sample size (*n*) for each experimental group/condition, given as a discrete number and unit of measurement
☐	☒	A statement on whether measurements were taken from distinct samples or whether the same sample was measured repeatedly
☐	☒	The statistical test(s) used AND whether they are one- or two-sided<br>*Only common tests should be described solely by name; describe more complex techniques in the Methods section.*
☒	☐	A description of all covariates tested
☐	☒	A description of any assumptions or corrections, such as tests of normality and adjustment for multiple comparisons
☐	☒	A full description of the statistical parameters including central tendency (e.g. means) or other basic estimates (e.g. regression coefficient) AND variation (e.g. standard deviation) or associated estimates of uncertainty (e.g. confidence intervals)
☐	☒	For null hypothesis testing, the test statistic (e.g. *F*, *t*, *r*) with confidence intervals, effect sizes, degrees of freedom and *P* value noted<br>*Give P values as exact values whenever suitable.*
☒	☐	For Bayesian analysis, information on the choice of priors and Markov chain Monte Carlo settings
☒	☐	For hierarchical and complex designs, identification of the appropriate level for tests and full reporting of outcomes
☐	☒	Estimates of effect sizes (e.g. Cohen's *d*, Pearson's *r*), indicating how they were calculated

Our web collection on statistics for biologists contains articles on many of the points above.

Software and code

Policy information about availability of computer code

Data collection	Data were collected by querying the Foundation Medicine database, which is accessible through the Foundation Insights portal.
Data analysis	Statistical tests and computation were carried out using Python 2.7 (Python Software Foundation) or R 4.1.1 (R Foundation for Statistical Computing).

For manuscripts utilizing custom algorithms or software that are central to the research but not yet described in published literature, software must be made available to editors/reviewers. We strongly encourage code deposition in a community repository (e.g. GitHub). See the Nature Research guidelines for submitting code & software for further information.

Data

Policy information about availability of data

All manuscripts must include a data availability statement. This statement should provide the following information, where applicable:

- Accession codes, unique identifiers, or web links for publicly available datasets
- A list of figures that have associated raw data
- A description of any restrictions on data availability

The data are derived from clinical samples. Approval for this study, including a waiver of informed consent and a HIPAA waiver of authorization, was obtained from the Western Institutional Review Board (protocol no. 20152817) prior to study conduct. The Institutional Review Board granted a waiver of informed consent under 45 CFR § 46.116 based on review and determination that this research meets the following requirements: (i) the research involves no more than minimal risk to subjects; (ii) the research could not practicably be carried out without the requested waiver; (iii) the waiver will not adversely affect the rights and welfare of the subjects. The variant call data used in this study are provided in Supplementary Data 1; other data necessary to verify the reported results are provided as Source Data. Patients did not consent to the release of underlying sequence data. Variant actionability was categorized according to OncoKB (https://www.oncokb.org/).

Field-specific reporting

Please select the one below that is the best fit for your research. If you are not sure, read the appropriate sections before making your selection.

☒ Life sciences ☐ Behavioural & social sciences ☐ Ecological, evolutionary & environmental sciences

For a reference copy of the document with all sections, see nature.com/documents/nr-reporting-summary-flat.pdf

Life sciences study design

All studies must disclose on these points even when the disclosure is negative.

Sample size	No sample size calculation was performed. This study includes data from all sarcoma specimens analyzed by Foundation Medicine between 2012–2018.
Data exclusions	Tissues with insufficient tumor or DNA yield were excluded; this exclusion was pre-established.
Replication	Because the data are from a very large cohort of patient samples, the analyses cannot be replicated.
Randomization	This is an observational study, so no randomization was performed.
Blinding	No participants were allocated to groups.

Reporting for specific materials, systems and methods

We require information from authors about some types of materials, experimental systems and methods used in many studies. Here, indicate whether each material, system or method listed is relevant to your study. If you are not sure if a list item applies to your research, read the appropriate section before selecting a response.

Materials & experimental systems

n/a	Involved in the study	
☒	☐	Antibodies
☒	☐	Eukaryotic cell lines
☒	☐	Palaeontology
☒	☐	Animals and other organisms
☐	☒	Human research participants
☒	☐	Clinical data

Methods

n/a	Involved in the study	
☒	☐	ChIP-seq
☒	☐	Flow cytometry
☒	☐	MRI-based neuroimaging

Human research participants

Policy information about studies involving human research participants

Population characteristics	Patients' median age was 52 years (range < 1 to > 88 years) and 53.3% were female. Diagnoses are presented in Fig. 1a.
Recruitment	Participants were not directly recruited. All genomic profiling (GP) was performed in the course of routine clinical management; the decision to pursue GP was at the discretion of treating physicians, in consultation with patients.
Ethics oversight	Western IRB and Memorial Sloan Kettering Cancer Center IRB

Note that full information on the approval of the study protocol must also be provided in the manuscript.
